# Implementing response-adaptive designs when responses are missing: Impute or ignore?

**DOI:** 10.1177/09622802251366843

**Published:** 2025-08-29

**Authors:** Mia S Tackney, Sofía S Villar

**Affiliations:** MRC-Biostatistics Unit, University of Cambridge, UK

**Keywords:** Multi-armed bandit, missing data, response adaptive procedures, sequential allocation, imputation, truncation

## Abstract

Missing data is a widespread issue in clinical trials, but is particularly problematic for digital health interventions where disengagement is common and outcomes are likely to be missing not at random (MNAR). Trials that use response-adaptive designs need to handle missingness online and not simply at the end of the trial. We propose a novel online imputation strategy which allows previous imputations to be re-imputed given updated estimates of success probabilities. We additionally consider: (i) truncation of deterministic algorithms to prevent extreme realised treatment imbalance and (ii) changing the random component of semi-randomised algorithms. Through a simulation study based on a trial for a digital smoking cessation intervention, we illustrate how the strategy for handling missing responses can affect the exploration-exploitation tradeoff and the bias of the estimated success probabilities at the end of the trial. In the settings explored, we found that the exploration-exploitation tradeoff is affected particularly when arms have very different rates of missingness and we identified combinations of response-adaptive designs and missingness strategies that are particularly problematic. Further, we show that estimated success probabilities at the end of the trial can be biased not only due to optimistic sampling, but potentially also due to an MNAR missingness mechanism.

## 1. Introduction

### 1.1. Designs for digital health interventions

Missing data is a common issue in clinical trials and can complicate the analysis and interpretation of results.^1^ In trials to evaluate digital health interventions, missing data are particularly pervasive. Digital health interventions typically promote healthy behaviors such smoking cessation or increased physical activity^2^ via text messaging, mobile phone applications (apps) or other forms of digital technology. Here, missingness rates may be particularly high for treatment arms that require more engagement, as participants may find that the burden of engaging exceeds the benefits.^3^ Participants may also habituate to the intervention, leading to reduced engagement.^4^ Further, in these settings, outcomes are likely to be missing not at random (MNAR); for example, participants in a trial for a smoking cessation intervention may engage less if they are still smoking.^5^

Trials for a digital health intervention often use some form of response-adaptive design, where treatment allocation is adapted based on the accumulating data during the trial. Recent examples that use response-adaptive designs include a trial for automated messaging to provide support and information for mental health^6^ and a trial for self-guided interventions to reduce psychological stress.^7^ Response-adaptive designs have a close connection with the multi-armed bandit problem (MABP), which is a framework for allocating treatments to participants in ways which balance two conflicting objectives: firstly, the objective to maximise *learning* about the most effective treatment and to *explore* the treatment space; secondly, the objective to maximise the total immediate *earning* and to *exploit* the current best treatment. There is a dilemma between *exploration* and *exploitation* or between *learning* and *earning*: allocating the treatment that is currently best performing may mean that the discovery of a better performing is never realised, on the other hand, exploring all treatments may mean that the total reward is not maximised. While response-adaptive designs for bandit problems have typically been deterministic, there has been increased interest in response-adaptive randomisation, particularly for applications to clinical trials.^8^

Response-adaptive designs are particularly suited for trials for digital health interventions. Participants typically become available sequentially, and the digitalized nature of the trial delivery allows treatments to be delivered and responses to be collected in real time.^9^ Further, as response-adaptive designs skew allocation towards a more effective treatment, they may help to reduce missing responses by skewing allocation to the arm that has a higher retention rate. In spite of this potential for response-adaptive designs to reduce missingness, the implementation of these designs when responses are missing is not straightforward. The responses of the current and past participants are needed to determine the allocation of the next participant. Therefore, when responses are missing, modification is required in the *design* and missing data is an issue for both the design and analysis stages of the trial.

### 1.2. Motivating example: The iCanQuit trial for smoking cessation

We focus on a two-arm, binary response setting motivated by a trial comparing two digital interventions for smoking cessation: *iCanQuit*, an Acceptance and Committment Therapy-based smoking cessation app (experimental arm), versus *QuitGuide*, a US Clinical Practice Guidelines-based app which focuses on avoiding triggers (control arm).^10^ The primary outcome was self-reported 30-day point prevalence abstinence (PPA) at 12 months after randomisation. The trial recruited 2415 participants between May 2017 and September 2018. They found that iCanQuit participants had 1.49 times higher odds (with 95% confidence interval [1.22,1.83] ) of quitting smoking compared to QuitGuide participants. The success probability for QuitGuide was 0.282 and the success probability of QuitGuide was 0.211. There was a 87.2% retention rate for the primary outcome, with 14.3% of responses missing in the experimental arm and 11.2% of responses missing in the control arm. When missing outcomes were imputed as smokers, the effect size was found to be similar (odds ratio of 1.40 with 95% confidence interval given by [1.14,1.71] ).

Participants were randomised in a 1:1 ratio using permuted blocks of size 2, 4 and 6, which were stratified by demographic and smoking-related variables. We examine, through a simulation study, the potential consequences of using a response-adaptive design, which would be feasible via adaptation on a shorter-term response. For example, this could be a 7-day point prevalance abstinence after randomisation, which, for simplicity, we assume to be perfectly correlated with the primary outcome for the purpose of the simulation. Response-adaptive methods such as BRAR have been used previously in smoking cessation trials such as in Faseru et al.^11^

### 1.3. Response-adaptive designs

We now describe the notation and framework for response-adaptive designs. When a participant i enrolls in a trial, they are assigned to treatment k∈{0,1} , where k=0 denotes the control arm and k=1 denotes the experimental arm. The binary treatment indicator $a_{i}$ takes value k when arm k is assigned to participant i . We denote by I={1,2,…,n} the set of participants who enroll in the trial. We assume that the response $y_{i}$ is binary and observed immediately, with $y_{i}=1$ denoting a favourable response (‘success’) and $y_{i}=0$ denoting an unfavourable response (‘failure’). We partition I into the set of participants who are assigned to arm 0, $I_{0}=\{m_{1},m_{2},\ldots ,m_{n_{0}}\}$ , and the set of participants who are assigned arm 1, $I_{1}=\{q_{1},q_{2},\ldots ,q_{n_{1}}\}$ , where $n_{0}+n_{1}=n$ . We can then partition the responses $y_{i}$ into a vector of responses for arm 0, $y_{0,t}=y_{m_{t}}$ for $t\in \{1,2,\ldots ,n_{0}\}$ , and a vector of responses for arm 1, $y_{1,t}=y_{q_{t}}$ for $t\in \{1,2,\ldots ,n_{1}\}$ .

The response $y_{k,t}$ is a realisation of the $\text{Bernoulli}(p_{k})$ distribution with unknown parameter $p_{k}$ depending on the assigned treatment k . The random variables $S_{k,t}$ and $F_{k,t}$ are the total number of successes and failures when t participants are assigned to arm k , respectively, with realisations given by $s_{k,t}=\sum_{j=1}^{t}y_{k,j}$ and $f_{k,t}=\sum_{j=1}^{t}(1-y_{k,j})$ . Designs which adopt a Bayesian approach assign a Beta( $s_{k,0},f_{k,0}$ ) prior distribution to each parameter $p_{k}$ . As participants enter the trial and data accrues, the posteriors for $p_{k}$ are Beta( $s_{k,0}+s_{k,t},f_{k,0}+f_{k,t})$ distributions, with posterior mean for success probability given by $\left(s_{k,0}+s_{k,t})/(s_{k,0}+s_{k,t}+f_{k,0}+f_{k,t}\right)$ .

There is an extensive literature on proposed designs that assign treatment to a newly enrolled participant, given the state $X_{t}=(s_{k,0}+S_{k,t},f_{k,0}+F_{k,t})$ . These designs allow allocations to be guided by responses according to certain criteria. These principles can be positioned on a number of spectra, such as deterministic versus randomised, myopic versus non-myopic and power-oriented versus patient-centred. We describe several response-adaptive designs to cover a range of characteristics along these spectra. Randomised designs are represented the by allocation probability $\pi_{k,t}=P(a_{k,t}=1)$ that participant t is allocated to treatment k . In deterministic and semi-randomised designs, an allocation index $I_{k,t}$ is computed for each arm and the participant t is allocated to the arm k with highest index. For a detailed overview of the literature, we refer readers to Robertson et al.^8^ for response-adaptive randomisation, and Villar et al.^12^ and Jacko^13^ for non-randomised designs.

**Equally randomised designs**

- Fixed randomisation (FR): $\pi_{k,t}=0.5$ in a two-armed setting. The randomisation probability is fixed and unaffected by the accruing responses.
- Permuted block randomisation (PBR): participants are randomised to treatments in blocks of size b to achieve exact balance within each block, where b can vary.

**Response-adaptive randomised designs**

- Bayesian response adaptive randomisation (BRAR): $\pi_{k,t}$ is a draw from the Beta $\left(s_{k,0}+s_{k,t},f_{k,0}+f_{k,t}\right)$ distribution. The allocation probability for arm k is proportional to the posterior probability that arm k has the highest successes probability.^14^ We note that there are regularised versions of this algorithm (see, e.g. Thall and Wathen^15^). BRAR is the most commonly implemented response-adaptive design used in practice^16^ in clinical trials.
- Neyman allocation: $\pi_{1,t}=\sqrt{{\hat{p}}_{1}(1-{\hat{p}}_{1})}/(\sqrt{{\hat{p}}_{1}(1-{\hat{p}}_{1})}+\sqrt{{\hat{p}}_{0}(1-{\hat{p}}_{0})})$ is an optimal allocation method which aims to maximise power for a *Z*-test given a fixed sample size. This is an example of a power-oriented response-adaptive design that can be implemented with a randomised procedure to target an estimated optimal proportion.^17^

**Deterministic response-adaptive designs**

- Gittins index (GI): $I_{k,t}=G(X_{k,t})$ , where G(⋅) denotes a Gittins index which can be found in tables provided by Gittins et al.^18^ The Gittins index provides a computationally tractable solution to the MAB problem in the infinite-horizon setting with discounted responses,^19^ which is otherwise solved via dynamic programming. This is an example of a non-myopic design, which favours exploration over exploitation.
- Current belief (CB): $I_{k,t}=(s_{k,0}+S_{k,t})/(s_{k,0}+S_{k,t}+f_{k,0}+F_{k,t})$ . Participants are allocated the treatment with the highest posterior probability of success. This is a myopic design which lends itself to an early selection of an arm.

**Semi-randomised response-adaptive designs**

Semi-randomised versions of GI and CB perturb the index with a random component, $Z_{t}\cdot \lambda_{k}(t)$ , where $Z_{t}$ is a deviate from an exponential distribution with parameter 1/K , where K is the number of arms, and $\lambda_{k}(t)$ is defined as follows:

(1) $$\lambda_{k}(t)=\frac{K}{s_{k,0}+f_{k,0}+S_{k,t}+F_{k,t}}.$$

The indices are defined as:

- Randomised Gittins index (RGI) $I_{k,t}=G[s_{k,0}+S_{k,t}][\;f_{k,0}+F_{k,t}]+Z_{t}\cdot \lambda_{k}(t)$ .
- Randomised belief index (RBI): $I_{k,t}=\frac{s_{k,0}+S_{k,t}}{s_{k,0}+S_{k,t}+f_{k,0}+F_{k,t}}+Z_{t}\cdot \lambda_{k}(t)$ .

**Regularisation**

When using response-adaptive designs, there is a risk of extreme imbalance in treatment allocation both throughout and at the end of the trial. When there are very few or no participants assigned to arm k , estimation of $p_{k}$ becomes difficult or even impossible. Treatment imbalance can be further exacerbated when responses are missing. Regularisation refers to methods which mitigate extreme imbalance in treatment arms in response-adaptive designs.^20,21^ This includes incorporating a *burn-in* period where participants are allocated to treatments with equal probability at the beginning of a trial, and *clipping*, which bounds randomisation probabilities within specified limits. We propose *truncation* for deterministic algorithms (such as GI and CB) with a similar aim to that of clipping for randomised algorithms. In a truncated design, to assign a treatment to participant i+1 , we compute the proportion of participants allocated to arm 1, which we denote $p^{*}(i)$ :

(2) $$p^{*}(i)=\frac{\sum_{j=1}^{i}a_{j}}{i}.$$

If $0.1<p^{*}(i)<0.9$ , the allocation procedure proceeds as usual. If $p^{*}(i)>0.9$ , we allocate arm 0 to participant i+1 . If $p^{*}(i)<0.1$ , we allocate arm 1 to participant i+1 .

### 1.4. Missing responses for fully sequential designs

Fully sequential designs typically assume that the response is observed immediately. However, in trials for digital health interventions, some responses will be missing due to a variety of possible causes, such as participants declining to respond to specific questions, habituation and disengagement, loss of connectivity or technical errors. Therefore, the implementation of fully sequential designs require modification to handle missing data *online as the data accrues*, rather than at the end of the study. We denote by $y_{i}^{\mathrm{obs}}$ an observed response and $y_{i}^{\mathrm{mis}}$ a missing response. The vector $y_{i}$ can be partitioned as $y_{i}=[y_{i}^{\mathrm{obs}},y_{i}^{\mathrm{mis}}]$ .

We denote by $r_{i}$ a missing data indicator such that:

$$r_{i}=\left\{ \begin{array}{ll} 1 & \text{if }y_{i}\text{ is missing}\\ 0 & \text{if }y_{i}\text{ is observed} \end{array}\right.$$

Analogously, we denote by $r_{k,t}$ the missingness indicator for $y_{k,t}$ and we can partition the response for arm k as $y_{k,t}=[y_{k,t}^{\mathrm{obs}},y_{k,t}^{\mathrm{mis}}]$ . We denote by $n^{\mathrm{obs}}$ the total number of observed responses in the trial, and denote by $n_{k}^{\mathrm{obs}}$ the total number of observed responses in arm k .

The missing data mechanism is characterised by the conditional distribution of $r_{i}$ given the data. The data consist of the incomplete response $y_{i}$ and fully observed data $x_{i}$ , which includes fully observed treatment assignment $a_{i}$ and possibly fully observed covariates $z_{i}$ . While we do not consider covariates in this paper, we discuss them briefly here to illustrate the missing data mechanisms. Specifically, according to Rubin’s taxonomy of missing data mechanisms,^22^ responses are:

- **Missing completely at random (MCAR)** if $P(r_{i}=1|y_{i},x_{i})=c$ for some constant c which does not depend on any of the variables. The probability of an response being missing has no association with observed or unobserved variables;
- **Missing at random (MAR)** given the observed variables $x_{i}$ if $P(r_{i}=1|y_{i},x_{i})=P(r_{i}=1|x_{i})$ , meaning that the probability that a response is missing depends only on observed variables. In the specific case where $x_{i}=a_{i}$ , the data are missing at random given the assigned treatment;
- **Missing not at random (MNAR)** if, after conditioning on the observed variables, $r_{i}$ depends on the incomplete variable $y_{i}$ .

The missing data mechanisms are important as the validity of methods for handling missing data (i.e. whether estimators of population parameters are consistent and inferences are correct) depends on the nature of the mechanisms. There is an extensive literature on handling missing data in clinical trials, where methods can be broadly categorised as complete case analysis, likelihood-based approaches, weighting approaches such as inverse probability weighting (IPW) and imputation-based methods such as multiple imputation. We refer readers to key works such as Van Buuren,^23^ Kenward and Carpenter^24^ and Carpenter et al.^25^ for an introduction to these topics. The missing data literature for clinical trials largely focuses on settings where treatment assignment does not depend on response; therefore, missing data is typically not an obstacle for treatment assignment and is a problem for the final analysis of data at the end of the trial. When data are missing in the response-adaptive setting, there are several new considerations:

1. **Need to handle missingness *online* to determine treatment allocation**
  Assignment of treatment for the current participant depends on the history of assigned treatments and responses. If response for participant t−1 is missing, uncertainty is introduced in $s_{k,t-1}$ and $f_{k,t-1}$ and a decision needs to be made on how to compute the allocation index or allocation probability for participant t . Therefore, we see the issue of handling missing data in an *online* manner (or as the trial data accrues) as a design issue.
2. **Impact on the realised balance between learning and earning**.
  Due to the dependence of treatment assignment on the responses, missing data can impact the *realised* balance between exploration and exploitation for some designs and this may depend on how missing data is handled. For example, if responses from a particular arm are mostly missing, this can impact the extent to which that arm is explored. Alternatively, if successes are likely to be mostly missing, this may impact the extent to which the allocation is skewed to the *truly* superior arm (as the missing successes can bias the current estimate of superior arm). Further, high rates of missing responses can make extreme imbalance in treatment assignment more likely than under complete data for some response-adaptive design. The impact on realised balance can have consequences on the operating characteristics of the trial, such as Type I error, power and the expected number of successes.
3. **Several potential sources of bias for the final analysis.**
  Efficient unbiased estimation in trials with response-adaptive designs is challenging as the dependence between treatment assignment and response can induce a negative bias in the estimate of treatment effect in finite sample settings due to the *optimistic* sampling.^26–29^ Unbiased estimators have been proposed^30^; when data are MNAR, there is an additional source of bias.
4. **New complexities for sensitivity analyses**.
  When data are missing, analyses proceed by making an assumption about the missing data mechanism. Since it is typically not possible to verify missing data assumptions using the observed data alone, sensitivity analyses are encouraged to illustrate the robustness of results to other missing data mechanisms.^1^ For response-adaptive designs, sensitivity analyses are not straightforward as considering a different missing data mechanism will have implications not only for responses but also the assigned treatments.

In this article, we focus on the first three issues outlined above. We build on recent work by Chen et al.^31^ who conducted an extensive simulation study which compared complete cases versus single imputation for a number of response-adaptive designs when responses are MCAR or MAR given the treatment arm. We propose a novel imputation strategy, to be used under an MCAR or MAR assumption, specifically in the sequential allocation setting. Further, we illustrate the impact of responses that are MNAR on the balance between learning/earning and optimistic sampling bias, which has not yet been illustrated in the context of response-adaptive designs.

In Section 2, we describe the missing data strategies for response-adaptive designs. In Section 3, we outline our simulation study which compares using complete cases and three single imputation approaches for a number of response-adaptive designs when responses are MCAR, MAR given treatment arm, or MNAR. Results are presented in Section 4. In Section 5, we outline key findings from the simulation study and highlight a number of areas for future work at the intersection between response-adaptive designs and missing data.

## 2. Strategies for implementing response-adaptive designs when data are missing

We describe two general classes of methods for computing allocation probabilities/indices when responses are missing: complete cases, which ‘ignore’ the missing responses, and single imputation, which replaces the missing responses with a single value. We describe several single imputation strategies, some of which are appropriate under a MAR assumption and some of which are appropriate under an MNAR assumption. We propose a novel imputation strategy under the MAR assumption. We also consider further modifications for response-adaptive algorithms when responses are missing, which include truncation and changing the random component for semi-randomised algorithms.

**Adapt with estimates based on complete cases**

When using complete cases, allocation probabilities/indices (such as those introduced in Section 1.3), are computed based on the numbers of successes $s_{k,t}$ failures $f_{k,t}$ from the observed responses $y_{k,t}^{\mathrm{obs}}$ and priors $s_{k,0},f_{k,0}$ .

**Adapt with estimates based on single imputation**

We describe three methods of imputing a missing response with a single value based on an assumed missing data mechanism. The first two approaches provide stochastic imputations, while the third involves a deterministic imputation when there is a strong assumption about an MNAR mechanism.

1. Single impute current (when responses are MCAR or MAR):
  This approach is referred to as ‘mean imputation’ in Chen et al.^31^ and its performance was compared to complete cases in an extensive simulation study.
  - Suppose that participant t is allocated to treatment k and their response $y_{k,t}$ is missing. We generate an imputation:
(3) $$y_{k,t}^{\mathrm{imp}}∼\text{Bernoulli}(\;{\hat{p}}_{k,t-1})$$
    where ${\hat{p}}_{k,t-1}$ is the maximum likelihood estimate (MLE) of the success probability for arm k , given the information up to time t−1 : $s_{k,t-1}/(s_{k,t-1}+f_{k,t-1})$ . If there is a missing response at an early stage of the trial before an MLE can be computed, we proceed with an imputation $y_{k,t}^{\mathrm{imp}}∼\text{Bernoulli}(0.5)$ , reflecting an apriori success probability of 0.5 in each arm.
    We note that there may be other natural choices for imputing under a MAR mechanism, such as the posterior mode. One could also use a proper imputation, which would entail drawing from the posterior distribution of the success probability and generating an outcome (success or failure) from it.
  - We then set the vector of responses as $y_{k,t}=[y_{k,t}^{\mathrm{obs}},y_{k,t}^{\mathrm{imp}}]$ and compute allocation probabilities/indices using $y_{k,t}$ and update MLEs ${\hat{p}}_{k,t}$ . The imputed value $y_{k,t}^{\mathrm{imp}}$ is treated as fixed (i.e. these values are not imputed again).
2. Single impute backward (when responses are MCAR or MAR):
  We note that in the single impute current approach, imputations that are generated early on in the trial are based on estimates of $p_{k}$ that may suffer from considerable bias.^30^ To overcome this limitation, we propose a new approach where imputations at time t are imputed again for $t^{\prime }<t$ , given that more data has accrued and estimates of $p_{k}$ are improved.
  - Suppose that participant t is allocated to treatment k and their response $y_{k,t}$ is missing, and suppose that $y_{k,t}^{\mathrm{mis}}$ is the vector of length q of all missing responses up until time t . We generate imputations for all q participants that have missing responses up until time t using the MLE:
(4) $$y_{k,t}^{\mathrm{imp}}=[\omega_{1},\omega_{2},\ldots ,\omega_{q}]$$
    where $\omega_{i}∼\text{Bernoulli}(\;{\hat{p}}_{k,t-1})$ for 1≤i≤q .
  - We then set the vector of responses as $y_{k,t}=[y_{k,t}^{\mathrm{obs}},y_{k,t}^{\mathrm{imp}}]$ and compute allocation probabilities/indices and updated estimates ${\hat{p}}_{k,t}$ . The imputed values $y_{k,t}^{\mathrm{imp}}$ are discarded for $t^{\prime }<t$ and new imputations are generated.
  - If there is a missing response before an MLE can be computed, we proceed with an imputation $y_{k,t}^{\mathrm{imp}}∼\text{Bernoulli}(0.5)$ .
  We note that these imputations based on the MLE are used to compute the allocation indices/probabilities but are not necessarily recommended to be used in calculating the final estimates of success probabilities at the end of the trial.
3. Single imputation with an informed choice of constant (when responses are MNAR):
  In some settings, an informed assumption can be made that missing values take a specific value. For example, guidance on the analysis of smoking cessation trials recommend sensitivity analyses where missing responses are imputed as smokers.^5,32^ In the iCanQuit trial, a sensitivity analysis showed that the effect size was similar when using complete cases versus when missing outcomes were imputed as smokers. Thus, we consider a single imputation approach where missing responses are simply imputed with a constant value c :
(5) $$y_{k,t}^{\mathrm{imp}}=c$$
  for c∈{0,1} .
  We note that imputation under a MNAR assumption can take several forms and imputing missing values as smoking makes a particularly strong assumption. One may wish to impute under a weaker MNAR assumption, for example by drawing from an appropriate quantile of the posterior distribution of the success probability.

These approaches to handling missing responses online are illustrated in Figure 1.

**Figure 1.**
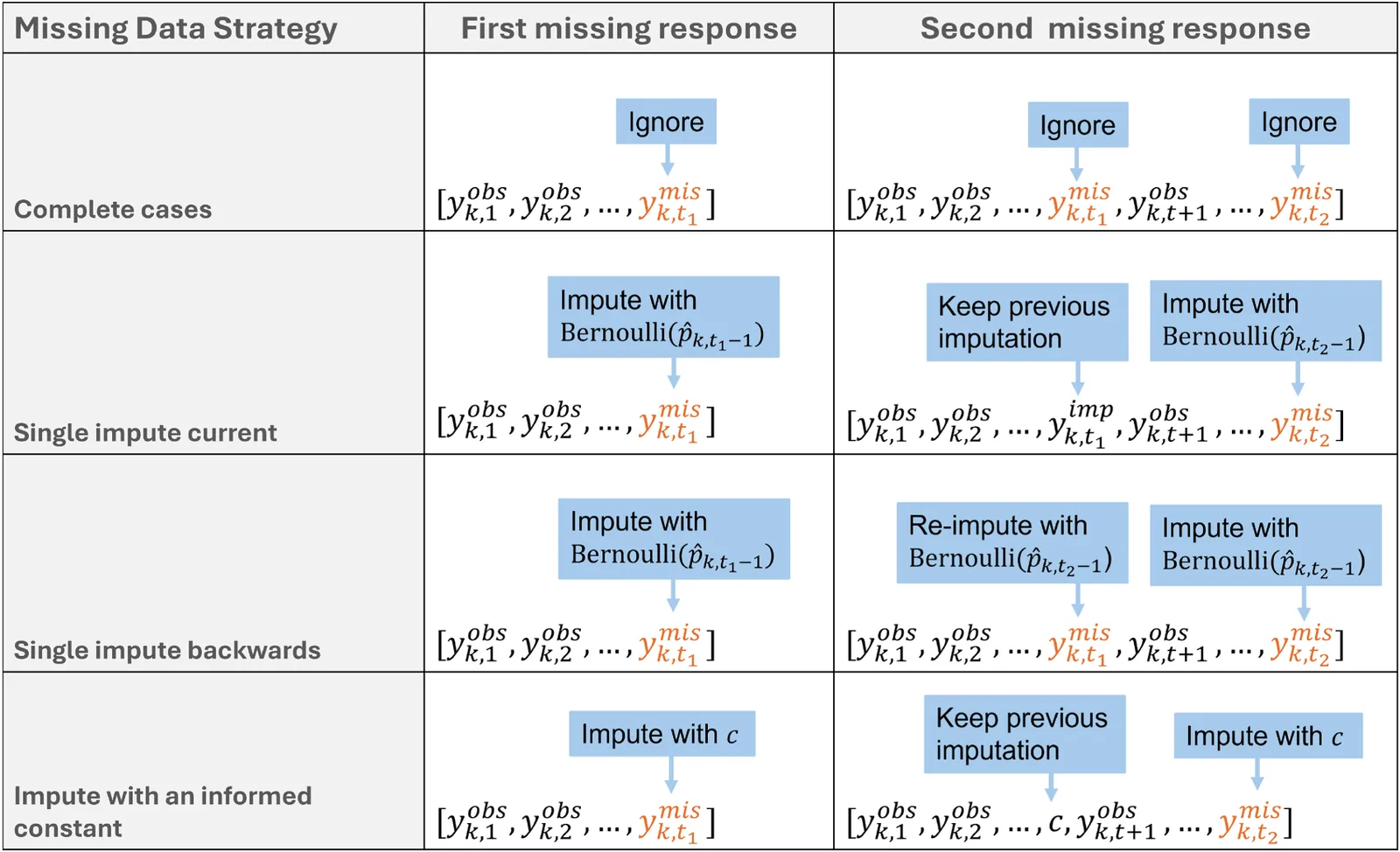
Illustration of how the first and second missing responses are handled online by different missing data strategies for response-adaptive algorithms. Note that, for single impute backward, the first missing response at time $t_{1}$ will be re-imputed as the estimates of success probabilities become updated. In contrast, imputations are kept fixed and do not become re-imputed in the other imputation approaches. To obtain allocation probabilities or indices, we compute $s_{k,t}$ and $f_{k,t}$ based on $y_{k,t}^{\mathrm{obs}}$ for complete cases and $y_{k,t}=[y_{k,t}^{\mathrm{obs}},y_{k,t}^{\mathrm{imp}}]$ for imputation approaches.

**Defining the random component for semi-randomised algorithms when data are missing**

Semi-randomised algorithms include a random component $Z_{t}\cdot \lambda_{k}(t)$ with $\lambda_{k}(t)$ as defined in Equation (1). There is ambiguity in how this component should be defined when data are missing. When data are fully observed or missing responses are imputed, we have that the denominator of $\lambda_{k}(t)$ is:

(6) $$s_{k,0}+f_{k,0}+t$$

reflecting the number of participants allocated to arm k as the prior number of successes and failures. When missing data are handled by complete cases, the denominator may be computed as:

(7) $$s_{k,0}+f_{k,0}+\sum_{j=1}^{t}(1-r_{k,j})$$

which reflects the number of *observed* responses for arm k as well as the prior number of successes and failures. Thus, missing responses in arm k can reduce the denominator of $\lambda_{k}(t)$ , which in turn increases the magnitude of the random component for arm k . We therefore consider an alternative definition of the random component when using complete cases, where $\lambda_{k}(t)$ is defined as in Equation (6), even if there are missing responses. We refer to this modified version of complete cases for RBI and RGI as *Complete cases nt* in the simulation study.

We compare the performance of these missing data strategies for response-adaptive designs in a simulation study in the next section.

## 3. Simulation study

Our simulation study, motivated by the iCanQuit trial, aims to compare the performance of the missing data strategies introduced in Section 2 under combinations of (i) different data generating mechanisms for the true response, including scenarios under the null and alternative hypotheses, (ii) different missing data mechanisms, including MCAR, MAR given treatment arm and MNAR mechanisms, and (iii) different response-adaptive designs, as described in Section 1.3. In particular, we wish to assess whether the choice of online missing data strategy can impact (i) the *realised* balance between exploitation and exploration and (ii) the observed bias of the final estimated success probabilities.

### 3.1. Data generating mechanism

#### 3.1.1. True response

The true binary response $y_{i}$ is generated according to $y_{i}∼\text{Bernoulli}(\theta_{i})$ , where

(8) $$\text{logit}(\theta_{i})=\beta_{0}+\beta_{1}a_{i}.$$

Here, $a_{i}$ denotes the treatment assignment for participant i and $\beta_{1}$ is the effect of treatment. We note that the success probabilities are given by $p_{k}=1/(1+\exp\;\left[-(\beta_{0}+\beta_{1}\cdot k)\right])$ . We consider four scenarios, outlined in Table 1, to cover a realistic range of settings for the iCanQuit trial:

- **Null**: success probability is 0.28 in both arms;
- **Null-low**: success probability is 0.07 in both arms;
- **Alternative**: success probability is 0.21 for QuitGuide and 0.28 for iCanQuit. These were the estimated success probabilities from the primary analysis;
- **Alternative-low**: the success probability is 0.07 for QuitGuide and 0.11 for iCanQuit. These were the hypothesized success probabilities which were used in the initial sample size calculations.^10^

**Table 1.** True response models in the simulation study: parameter values for equation (8) and probability of success in each treatment arm.

True data generating mechanisms	$\beta_{0}$	$\beta_{1}$	$p_{0}$	$p_{1}$
Null	− 0.95	0	0.28	0.28
Null-low	− 2.55	0	0.07	0.07
Alternative	− 1.3	0.36	0.21	0.28
Alternative-low	− 2.55	0.5	0.07	0.11

#### 3.1.2. Sample size

We simulate n=1622 participants. This was the sample size required for the iCanQuit trial to achieve 80% power for a 2-tailed significant difference between a success probability of 0.11 for iCanQuit and 0.7 for QuitGuide (the target recruitment was set higher for exploratory analyses).

#### 3.1.3. Missingness mechanism

The missingness indicator $r_{i}$ is generated as $r_{i}∼\text{Bernoulli(}\phi_{i})$ , where

(9) $$\text{logit}(\phi_{i})=\alpha_{0}+\alpha_{1}a_{i}+\alpha_{2}y_{i}.$$

We consider the following missing data mechanisms, summarised in Table 2, to cover a range of realistic patterns and magnitudes for missing responses in the iCanQuit trial:

- **Fully observed**: There is no missing data.
- **MCAR**: The probability of a missing response is 0.14.
- **MAR given the treatment arm**:
  – **MAR-T0-low**: The probability that a response is missing is 0.11 for iCanQuit and 0.14 for QuitGuide. These missingness rates are similar to what was observed in the 12-month outcome in the trial, however, we simulate a higher rate of missingness in the control arm rather than the experimental arm.
  – **MAR-T0-high**: The probability that a response is missing is 0.02 for iCanQuit and 0.5 for QuitGuide.
- **MNAR**:
  – **MNAR-Y0**: A success has probability 0.05 of being missing and a failure has probability 0.18 of being missing.
  – **MNAR-T0Y0**: For QuitGuide, a success has probability 0.05 of being missing and a failure has probability 0.18 of being missing. For iCanQuit, a success has probability 0.12 of being missing for QuitGuide and probability 0.03 of being missing for iCanQuit.

**Table 2.** Missing data mechanisms in the simulation study: parameter values for the missing data mechanism (equation (9)) and the probability of missingness for each combination of treatment assignment and response value.

Missing data mechanisms	$\alpha_{0}$	$\alpha_{1}$	$\alpha_{2}$	$P(r_{i}=1|a_{i}=0,y_{i}=1)$	$P(r_{i}=1|a_{i}=0,y_{i}=0)$	$P(r_{i}=1|a_{i}=1,y_{i}=1)$	$P(r_{i}=1|a_{i}=1,y_{i}=0)$
MCAR	− 1.8	0	0	0.14	0.14	0.14	0.14
MAR-T0-low	− 1.8	− 0.3	0	0.14	0.14	0.11	0.11
MAR-T0-high	0	− 4	0	0.5	0.5	0.02	0.02
MNAR-Y0	− 1.5	0	− 1.5	0.05	0.18	0.05	0.18
MNAR-T0Y0	− 1.5	− 0.5	− 1.5	0.05	0.18	0.03	0.12

### 3.2. Methods

Our simulation compares the performance of each of the following designs, introduced in Section 1.3:

- Randomised designs which target equal treatment allocation: fixed randomisation (FR) with $\pi_{k,t}=0.5$ ; permuted block randomisation (PBR) with block sizes of 2, 4 and 6 (no stratification factors are used);
- Randomised response-adaptive designs: Bayesian response-adaptive randomisation (BRAR); Neyman allocation, where treatment allocation is a function of estimated variances of the success probabilities. If estimated variances of $p_{0}$ or $p_{1}$ are zero (at the early stages of the trial), we assign treatment with equal probability^33^;
- Deterministic algorithms: Gittins index (GI) with discount factor 0.99 and current belief (CB).
- Semi-randomised algorithms: randomised Gittins index (RGI) and randomised belief index (RBI)

For each of the designs, we use four possible missing data approaches (complete cases, single impute current, single impute backward, impute zero), as described in Section 2. Further, specifically for RBI and RGI when complete cases are used, in addition to the default setting where the random component is defined as in Equation (7), we also show results when it is defined as an Equation (6), denoted *Complete cases nt*.

All designs begin with a permuted block of size 4 (two assignments to each arm), and we ensure that all four responses are observed. This prevents situations where missing responses at the beginning of the trial lead to extreme imbalance and prevent estimation of success probabilities. In Supplementary File 2, we provide results without this initialisation (i.e. there may be treatment imbalance in the first four participants, and they could have missing responses).

### 3.3. Estimands

The estimands include $p^{*}(1622)$ , the proportion of participants assigned to the truly superior arm (as defined in Equation (2)) at the end of the trial. Under the Null, we compute the proportion of participants assigned to arm 1, although arm 1 is not superior to arm 0. Additional estimands include the success probabilities $p_{0}$ and $p_{1}$ attained when n=1622 .

### 3.4. Performance measures

The performance measures include the mean of $p^{*}(1622)$ across 10,000 simulations, to assess whether the choice of missing data strategy can impact the balance between exploration and exploitation. The mean of the MLE of the success probabilities across 10,000 simulations, ${\hat{p}}_{0,1622}$ and ${\hat{p}}_{1,1622}$ , are additional performance measures. It is known that these estimates can be biased due to optimistic sampling; we assess whether the missing data strategies further impact this bias. For imputation strategies, we note that MLEs can be computed using complete cases only:

(10) $${\hat{p}}_{k,n}^{cc}=\frac{\sum_{t=1}^{n_{k}}y_{k,t}^{\mathrm{obs}}}{n_{k}^{\mathrm{obs}}},$$

or using both observed and imputed responses (using the final imputations, as opposed to partial or running imputations, for single impute backward):

(11) $${\hat{p}}_{k,n}^{\mathrm{imp}}=\frac{\sum_{t=1}^{n_{k}}y_{k,t}}{n_{k}}.$$

Further, when using BRAR, we additionally compute estimates of success probabilities using the inverse probability weighted (IPW) estimator.^30^ This is a bias-corrected estimator, which is defined as follows when using complete cases:

(12) $${\hat{p}}_{k,n}^{\mathrm{cc},\mathrm{IPW}}=\sum_{t=1}^{n_{k}}\frac{y_{k,t}^{\mathrm{obs}}}{\pi_{k,t}}/\sum_{t=1}^{n_{k}}\frac{1}{\pi_{k,t}},$$

and as below when using both observed and imputed responses (using the final imputations for single impute backward):

(13) $${\hat{p}}_{k,n}^{\mathrm{imp},\mathrm{IPW}}=\sum_{t=1}^{n_{k}}\frac{y_{k,t}}{\pi_{k,t}}/\sum_{t=1}^{n_{k}}\frac{1}{\pi_{k,t}}.$$

Each simulation setting is repeated 10,000 times. The simulation was performed in R version 4.3.1.^34^ Code to run the simulation is available on https://github.com/mst1g15/Response-Adaptive-Missing.

## 4. Results

We focus on the results for the Null scenario, where the success probability is 0.28 for both arms, and the Alternative scenario, where the success probability for the experimental arm is 0.28 and the success probability for the control arm is 0.21. Results for other settings are provided in Supplementary file 1.

### 4.1. Results for $p^{*}(1622)$

Figure 2 displays $p^{*}(1622)$ under the Null scenario. We omit results for fixed randomisation (as they are similar to results for the permuted block designs) and provide results for CB, CB-truncated and RBI in Figure A.1 in the Appendix. Each point indicates the mean of $p^{*}(1622)$ across 10,000 simulations for a particular combination of design, missing data scenario and missing data strategy. The error bars indicate 1.96× Monte Carlo (MC) standard error, but are too small to be seen in some cases.

**Figure 2.**
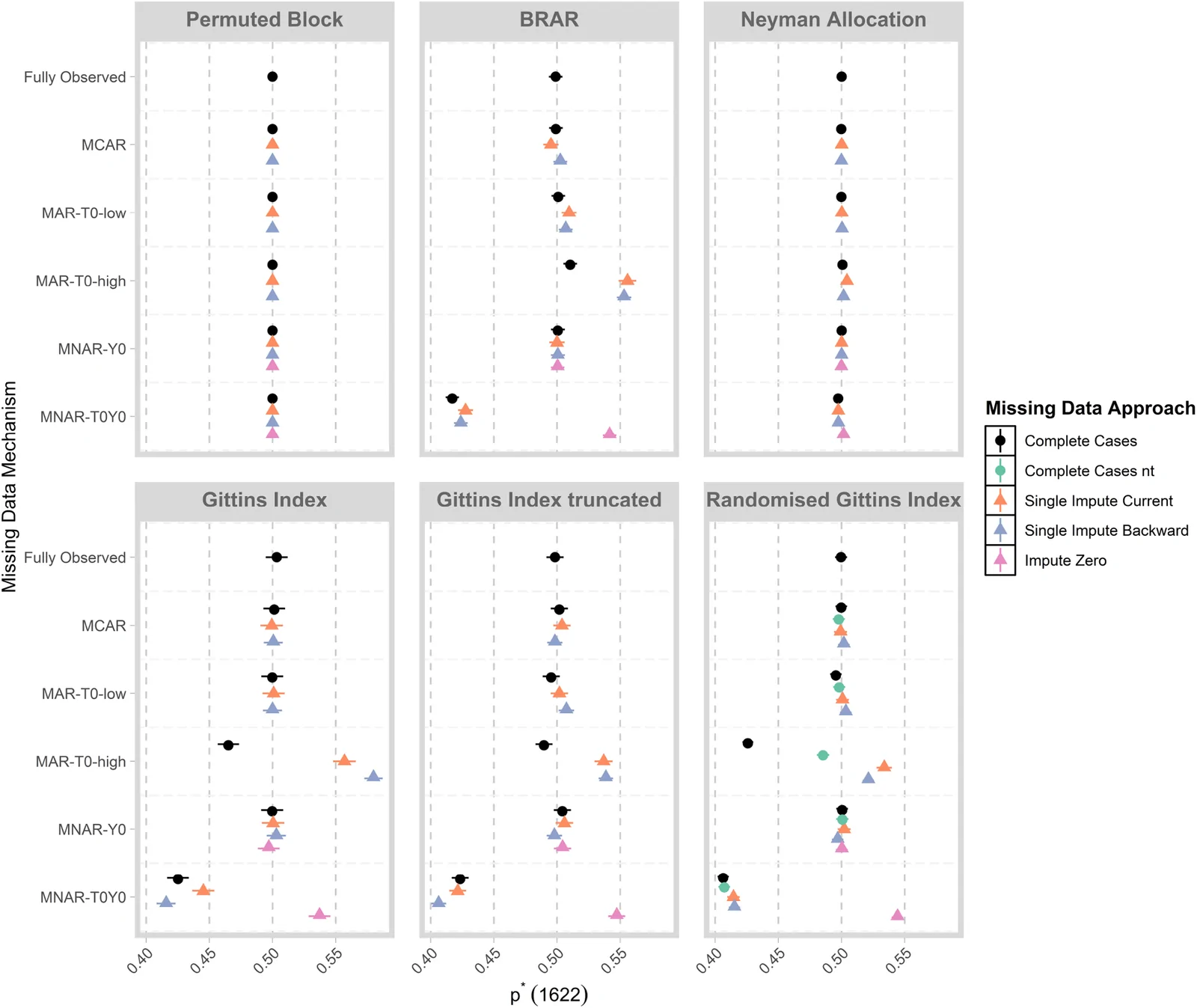
Mean of $p^{*}(1622)$ under the Null scenario for each combination of design, missing data mechanism and missing data strategy. Plots are based on 10,000 simulations and error bars indicate 1.96× MC standard error. *Complete cases-nt*: complete cases for semi-randomised algorithms, where randomised component is given by Equation (6). See Figure A.1 in the Appendix for additional designs.

Under the Null, when data are fully observed, all designs lead to equal allocation as expected, as neither arm is superior. The permuted block design and Neyman allocation lead to equal allocation under all missing data mechanisms and missing data strategies. For all other designs, when the missing data mechanism is the same in each arm (i.e. *MCAR, MNAR-Y0*), or the quantity of missing data is low (i.e. *MAR-T0-low*), we observe that the value $p^{*}(1622)$ is comparable to that obtained when data are fully observed. When there is a substantially different amount of missing data between arms (i.e. *MAR-T0-high, MNAR-T0Y0*) , several algorithms can lead to wrongly favouring an arm under the null.

Specifically, for *MAR-T0-high*, we observe that when complete cases are used in conjunction with designs that are geared towards exploration (e.g. GI, GI-truncated, RGI) more patients are assigned to the control arm. The tendency for these algorithms to favour the arm with more missing responses was noted by Chen et al.^31^ The extent of the selection can be reduced by using the truncated version of GI. Changing the random component to Equation (6) when using complete cases for RGI can also mitigate the selection of the control arm. Further, for BRAR, GI, GI-trunced and RGI, we observe that using single impute current and single impute backward leads to the selection of the experimental arm under MAR-T0-high. Some intuition can be gained by visualising the estimated values of $\hat{p_{0}}$ and $\hat{p_{1}}$ as sample size increases, as shown in Figure A.3 for BRAR and Figure A.4 for GI in the Appendix. We observe that single impute current and single impute backward lead to a larger negative bias for $p_{0}$ compared to $p_{1}$ . This makes arm 1 the *seemingly* better performing arm, resulting in greater allocation towards the experimental arm.

For MNAR-T0Y0, failures have a higher rate of missingness in for the control arm. Although the control arm has a greater rate of missingness, the MNAR mechanism means that the estimated success probability is greater for the control arm and it is selected for BRAR, GI, GI-truncated and RGI when used with complete cases, single impute current or single impute backward. When missing outcomes are imputed with zero, the experimental arm is favoured instead. This is because a small proportion of successes can be missing in the MNAR-T0Y0 setting, but impute-zero will impute all missing outcomes as failures, leading to selection in the opposite direction.

Figure 3 displays $p^{*}(1622)$ under the Alternative scenario. See Figure A.2 in the Appendix for additional designs. For all designs except for the permuted block design, the experimental arm is favoured, indicating selection of the superior arm. Missing data can lead to a reduction in $p^{*}(1622)$ , but not to the extent that the inferior arm is favoured in the simulation settings we have explored.

**Figure 3.**
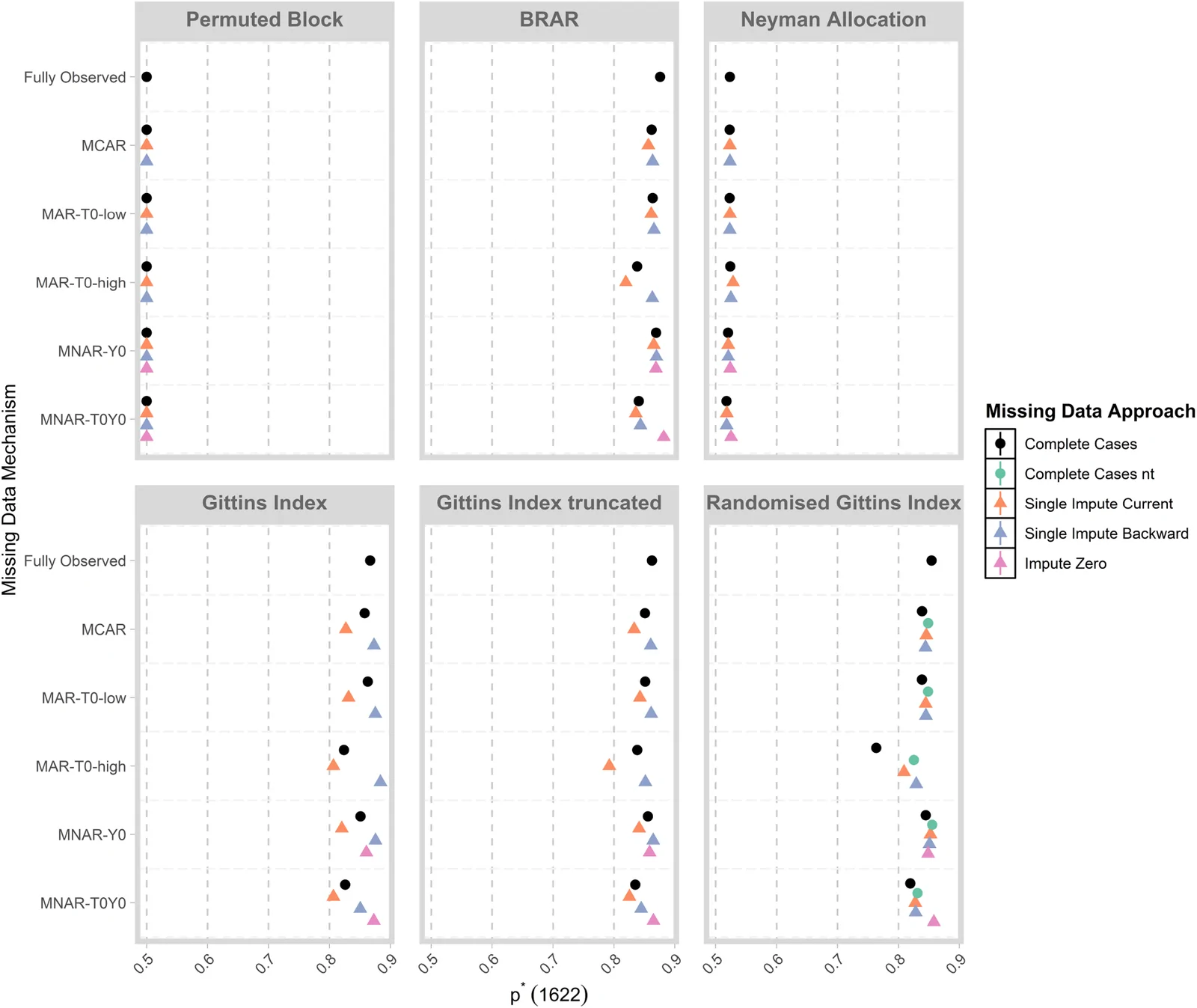
Distribution of $p^{*}(1622)$ under the Alternative scenario for each combination of design, missing data mechanism and missing data strategy. Plots are based on 10,000 simulations and error bars indicate 1.96× Monte Carlo (MC) standard error. *Complete-nt*: complete cases for semi-randomised algorithms, where randomised component is given by Equation (6). See Figure A.2 in the Appendix for additional designs.

Specifically, for BRAR, GI and GI-truncated, using single impute current can lead to a reduced value of $p^{*}(1622)$ , particularly under the *MAR-T0-high* scenario. For RGI, using complete cases particularly leads to reduced values of $p^{*}(1622)$ under the *MAR-T0-high* missing data mechanism. This is mitigated by changing the random component to Equation (6).

In Supplementary File 2, we display results when the design does not begin with a permuted block of size 4 with all responses observed. Here, we see that results for single impute current are very sensitive to specification of the initial part of the design.

We examine more closely the distribution of $p^{*}(1622)$ in selected algorithms and scenarios.

#### 4.1.1. BRAR

In Figure 4, we compare the distribution of $p^{*}(1622)$ over 10,000 simulations when data are complete versus under the *MAR-T0-high* mechanism for the Null and Alternative scenarios. We focus on the *MAR-T0-high* scenario as it leads to favouring the experimental arm under the null and reduction in $p^{*}(1622)$ under the alternative when single impute current is used. Under the Null, we observe a U-shaped distribution for single impute current, suggesting a greater proportion of simulations which have more extreme values of $p^{*}(1622)$ . Therefore, single impute current may not be a favourable strategy as it may lead to imbalance more frequently than complete cases or single impute backward. Under the Alternative, we observe that using single impute current can lead to a higher number of simulations where $p^{*}(1622)$ is equal to or very close to 1.

**Figure 4.**
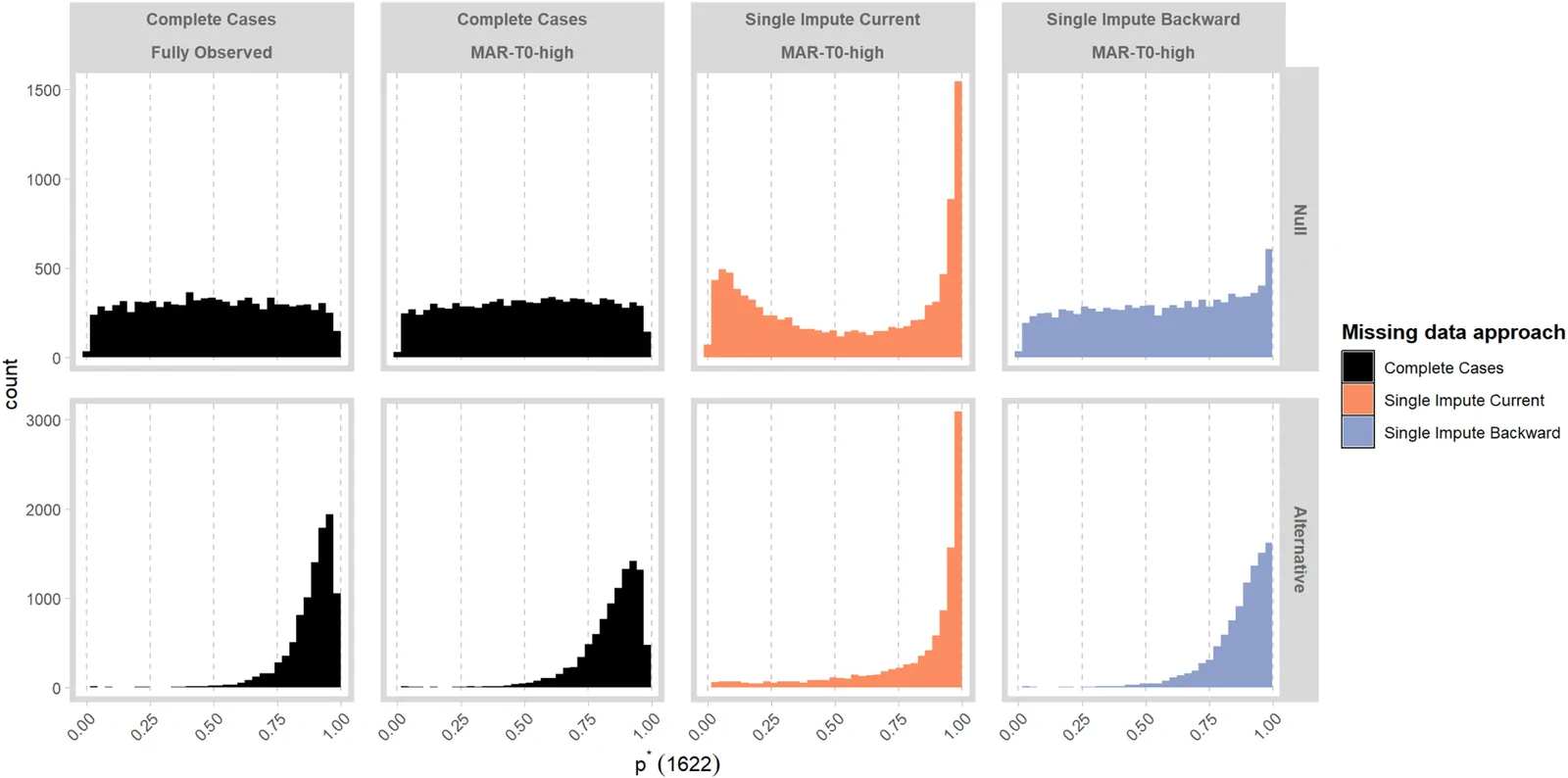
Distribution of $p^{*}(1622)$ under the Null scenario (top) and the Alternative scenario (middle and bottom) using BRAR when data are either fully observed or missing according to the *MAR-T0-high* mechanism. Missing data strategies used include complete cases, single impute current and single impute backward. Plots are based on 10,000 simulations.

#### 4.1.2. RGI

In Figure 5, we compare the distribution of $p^{*}(1622)$ for RGI over the 10,000 simulations when data are fully observed versus under the MAR-T0-high mechanism. Under the null, when complete cases are used, the distribution of $p^{*}(1622)$ is shifted to the left, revealing a preference towards the control arm. When single impute current is used, we observe that the distribution of $p^{*}(1622)$ appears to be bimodal. When single impute backward or complete cases nt is used, the distribution of $p^{*}(1622)$ appears to look more similar to what is observed when data are complete. Under the Alternative, we observe some extremely low values of $p^{*}(1622)$ when single impute current is used.

**Figure 5.**
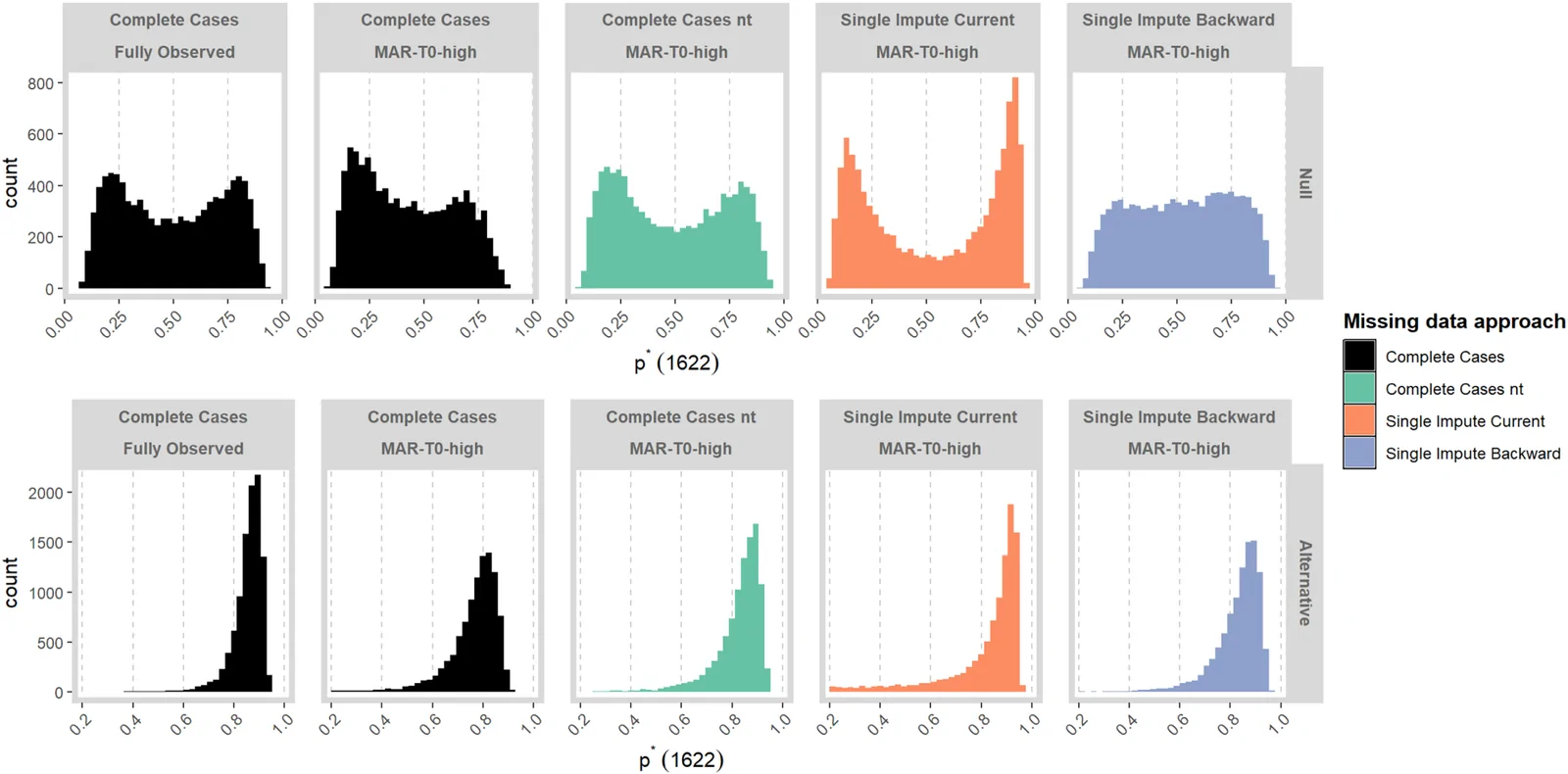
Distribution of $p^{*}(1622)$ under the Null (top) and Alternative (bottom) when data are *MAR-T0-high* when allocation proceeds with RGI. Missing data strategies include complete cases, complete cases nt, single impute current, single impute backward and Impute Zero. Plots are based on 10,000 simulations.

### 4.2. Results for the MLEs of $p_{0}$ and $p_{1}$

In Figure 6, we display the mean of MLEs of $p_{0}$ and $p_{1}$ for the permuted block design and BRAR under the Alternative scenario ( $\;p_{0}=0.5,p_{1}=0.881)$ . Additionally for BRAR, we display the estimates using the IPW-estimator which corrects for bias due to optimistic sampling.^30^ We focus on these designs as they are most commonly used in clinical trials. Results for other designs are provided in Supplementary File 1. When imputation is used, the estimates can be computed using complete cases only as in Equation (12) (indicated by a dot), or using both observed and imputed values as in Equation (13) (indicated by a star).

**Figure 6.**
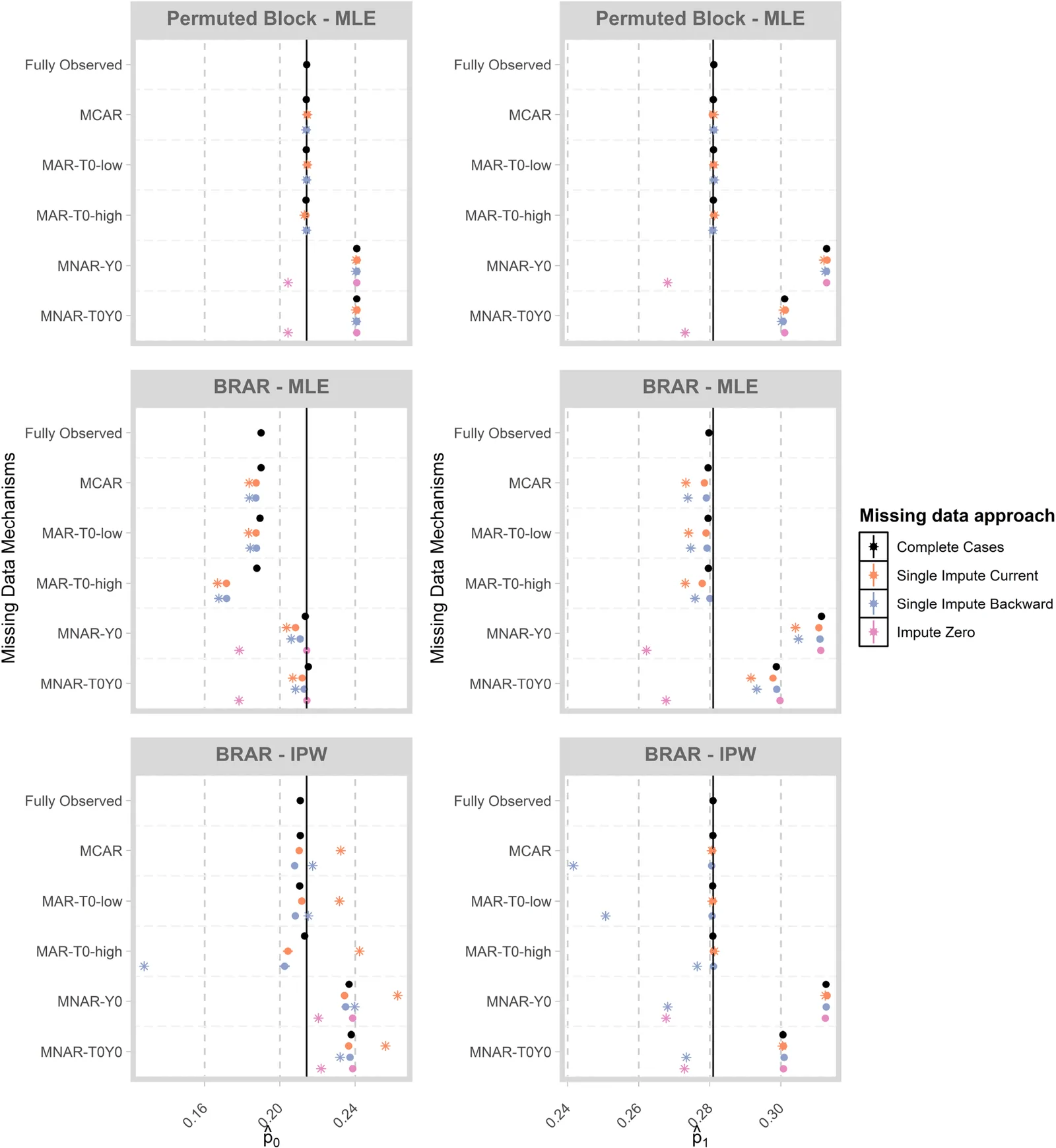
Mean of estimates of $p_{0}$ (left) and $p_{1}$ (right) across 10,000 simulations under the Alternative scenario when using the MLE for the permuted block design (top), the MLE for BRAR (middle) and the IPW estimator for BRAR (bottom). Error bars indicate 1.96 ± Monte Carlo standard error. For single impute current, single impute backwards and impute zero, the estimate can be computed using complete cases only, shown as a dot, or using both observed and imputed values, shown as a star. In several cases, the two estimates coincide.

For the permuted block design, we observe that estimates are generally unbiased when data are fully observed or missing under the MCAR, MAR-T0-low or MAR-T0-high mechanisms. Since we are estimating the success probabilities for each treatment, the MAR-T0-low and MAR-T0-high scenarios can be considered as MCAR *within each treatment group*. Under MNAR, we observe an upward bias unless missing outcomes are imputed as zero, in which case we observe a slight downward bias.

For BRAR, we observe negative bias in the MLE for $p_{0}$ , which is expected in finite sample settings for adaptive designs.^30^ In the MNAR settings, there is a combination of downward bias due to optimistic sampling as well as upward bias due to failures being missing. While the estimate for ${\hat{p}}_{0}$ appears unbiased under *MNAR-Y0* with complete cases, it is by coincidence that the downward bias due to optimistic sampling has a similar magnitude to the upward bias due to missing failures.

When the IPW-estimator is used for BRAR, we observe that the downward bias due to optimistic sampling is corrected when using estimates from complete cases under the MCAR, MAR-T0-low and MAR-T0-high mechanisms. Using the IPW-estimator in combination with imputed values can lead to bias, as the imputations are based on MLEs which are biased for adaptive designs. Further, we observe upward bias under the MNAR mechanism which is alleviated to some extent when imputing missing outcomes as zero. These results illustrate that, for response-adaptive designs, imputations based on the MLE can lead to biased results. Further, biases can be considerable when data are MNAR and analysis proceeds with complete cases or imputation with the MLE.

The estimates of $p_{0}$ and $p_{1}$ under the Null are provided in the Appendix in Figure A.5.

## 5. Discussion

We explored a number of strategies to handle missing data in the implementation of response-adaptive designs in a two-arm, binary response setting based on the iCanQuit trial. These included online imputation strategies, as well as modifications to algorithms such as truncation and changing the random component. We demonstrated the impact of the missing data mechanism and proportion of missing responses on (i) the realised balance between exploration and exploitation and (ii) the estimated success probabilities at the end of the trial. We summarise the findings below, with key considerations outlined in Table 3 and provide directions for future work.

**Table 3.** Design, implementation, and analysis considerations for response-adaptive designs with missing data from the icanQuit simulation study.

Stage of trial	Considerations
Design	Consider whether missing outcomes are likely and which missing data mechanisms are plausible.
	Regularisation can help prevent extreme imbalance, particularly for designs that lead to early selection (which can be exacerbated by missing data).
	For semi-randomised algorithms, consider how the random component should be defined if outcomes are missing.
Online implementation	Compute allocation probabilities/indices via complete cases or imputation. When there are substantially different missingness rates in the two arms, we saw that $p^{*}$ can be impacted:
	⋅ Single impute current is particularly sensitive to the initialisation of the design (i.e. burn-in and missing outcomes at the start of the trial).
	⋅ All missing data strategies can potentially lead to selection of an arm under the Null.
	⋅ Under the Alternative, single impute current can lead to reduced $p^{*}$ for several designs.
	⋅ If data are MNAR, imputing with the correct missing data assumption helps bring $p^{*}$ closer to value expected when data are fully observed.
Analysis	Bias due to optimistic sampling is corrected when using the IPW estimator (using complete cases) when data are fully observed, MCAR, or MAR. Imputations based on the MLE should not be used to compute the IPW estimator.
	If outcomes are MNAR, imputations based on a correct missing data assumption can help to reduce bias.

### 5.1. When does missing data affect the realised balance between exploration and exploitation?

When there are substantially different rates of missing data in the two arms, there can be a greater chance of realised imbalance in treatment allocation. In the iCanQuit trial, the experimental and control arms had 14.3% and 11.2% of outcomes missing, respectively; our simulations showed that $p^{*}(1622)$ is impacted minimally with these rates of missingness. However, at higher rates, for example where one arm has 50% of responses missing while the other arm has 2% of responses missing, the impact on treatment balance is large. In the Null scenario, several designs wrongly favour one arm and the selected arm depends on the choice of design and missing data strategy. Under the Alternative scenario, missing responses can lead to reduced values of $p^{*}(1622)$ , but in the settings explored, $p^{*}(1622)$ remains above 0.5 and the superior arm is still favoured overall. Specifically, our simulations demonstrated that:

- using complete cases together with algorithms that favour exploration can lead to the selection of the arm with greater missingness;
- using single impute current and single impute backwards when there are differential rates of missingness can lead to differential bias of the success probability of the two arms, which can drive selection of one arm;
- Single impute current is more prone to extreme imbalance in treatment allocation and is more sensitive to the initialisation of the design (see Supplementary File 2 for simulation results where there is no burn-in and initial responses can be missing).

We have shown that truncation can mitigate the impact of missing data on the imbalance for GI and CB by reducing the tendency of these designs to favour an arm early on, and further, changing the random component of the semi-randomised algorithms can improve the realised balance when complete cases are used.

A general recommendation at the design stage is to consider whether missingness rates may be different in the two arms to explore the potential impact on operating characteristics of the design through simulations. If differential missingness rates are suspected, we recommend **avoiding** the use of:

1. complete cases together with RBI and RGI. This can lead to lead to realised treatment imbalance under the Null as well as reduced proportion of participants allocated to the superior arm under the Alternative.
2. Single impute current with BRAR, GI-truncated and CB-truncated, as this can lead to selection Under the Null as well as reduced values of $p^{*}(1622)$ under the Alternative.

### 5.2. When does missing data induce additional bias in the MLE (i.e. beyond the optimistic sampling bias) at the end of the study?

The MLE at the end of the study for an adaptive design can have a small negative bias due to optimistic sampling, even when data are complete. Missing responses can change the direction and magnitude and bias of the MLE. In particular, when data are MNAR, we demonstrated that there is additional (upward or downward) bias in addition to the downward bias due to optimistic sampling.

When IPW estimator is used for BRAR, we note that the bias due to optimistic bias is generally corrected if outcomes are MCAR or MAR, but imputations generated from an MLE should generally not be used to compute the estimate. When data are MNAR, we note bias can be considerable for both the MLE and IPW estimators, unless imputations are correctly specified.

### 5.3. Future work

There are several important areas for future work. First, our simulations showed that different forms of regularisation can be helpful in reducing imbalance when there is risk of missing outcomes. We explored truncation for deterministic designs, where the proportion of participants allocated to the experimental arm are constrained between 0.1 and 0.9. Exploring other choices of thresholds for this truncation and their impact on operating characteristics is an area of future work.

Second, our simulations showed that differential rates of missingness in the two arms can have large implications for the realised balance between exploitation and exploration. Further, we showed that imputations generated from an MLE may not be ideal as they are generally biased due to optimistic sampling. Future work could investigate testing of differential rates of missing outcomes in arms and selecting an appropriate missing data strategy based on results of this test. This missing data strategy may involve an online imputation strategy which employs bias-corrected methods.^35^

Third, further work is needed in statistical inference when response-adaptive designs are used and outcomes are missing. For example, Type I error and power have been explored for the analysis of trials that use response-adaptive designs,^12,36,37^ but the impact of missing data on these operating characteristics require further exploration. An additional area of inference is assessing robustness of estimates to missing data assumptions. Sensitivity analyses are encouraged when responses are missing in clinical trials.^38^ When response-adaptive algorithms are used, sensitivity analyses are not straightforward as the consideration of an alternative missing data mechanism to the one actually assumed when designing the trial opens up different trajectories for how treatment allocations and subsequent responses could have unfolded. Thus, an approach to assessing robustness of missing data assumptions offline when response-adaptive designs are used is an important avenue of future work.

Fourth, exploration of missing data strategies for more complex settings which are characteristic of trials for digital health interventions are needed. In these settings, participants’ baseline characteristics or contextual information such as the time of day when the user is engaging with the intervention may be available. Such covariates may be predictive both of the response and the probability that the response is missing, in which case their inclusion in an imputation model will lead to improved imputations. Thus, incorporating covariates in imputation for response-adaptive procedures, as well as handling missing responses in covariate-adaptive randomisation procedures are important directions for future research. Further, exploration of more complex outcomes, such as longitudinal outcomes, categorical outcomes (e.g. questionnaire outcomes) or continuous outcomes (e.g. step count), as well as more complex treatment structures such as factorial designs, are much needed areas for further investigation due to their relevance for digital health interventions.

We focused on a selection of response-adaptive designs applied to the setting of digital health interventions. This investigation has shed light on the potential impact of missing data for other response-adaptive designs and applications to other types of interventions, and also highlighted several open questions and remaining challenges in this area.

## Supplemental Material

sj-pdf-1-smm-10.1177_09622802251366843 - Supplemental material for Implementing response-adaptive designs when responses are missing: Impute or ignore? Supplemental material, sj-pdf-1-smm-10.1177_09622802251366843 for Implementing response-adaptive designs when responses are missing: Impute or ignore? by Mia S Tackney and Sofía S Villar in Statistical Methods in Medical Research

sj-pdf-2-smm-10.1177_09622802251366843 - Supplemental material for Implementing response-adaptive designs when responses are missing: Impute or ignore? Supplemental material, sj-pdf-2-smm-10.1177_09622802251366843 for Implementing response-adaptive designs when responses are missing: Impute or ignore? by Mia S Tackney and Sofía S Villar in Statistical Methods in Medical Research

## Figures and Tables

**Figure 7.**
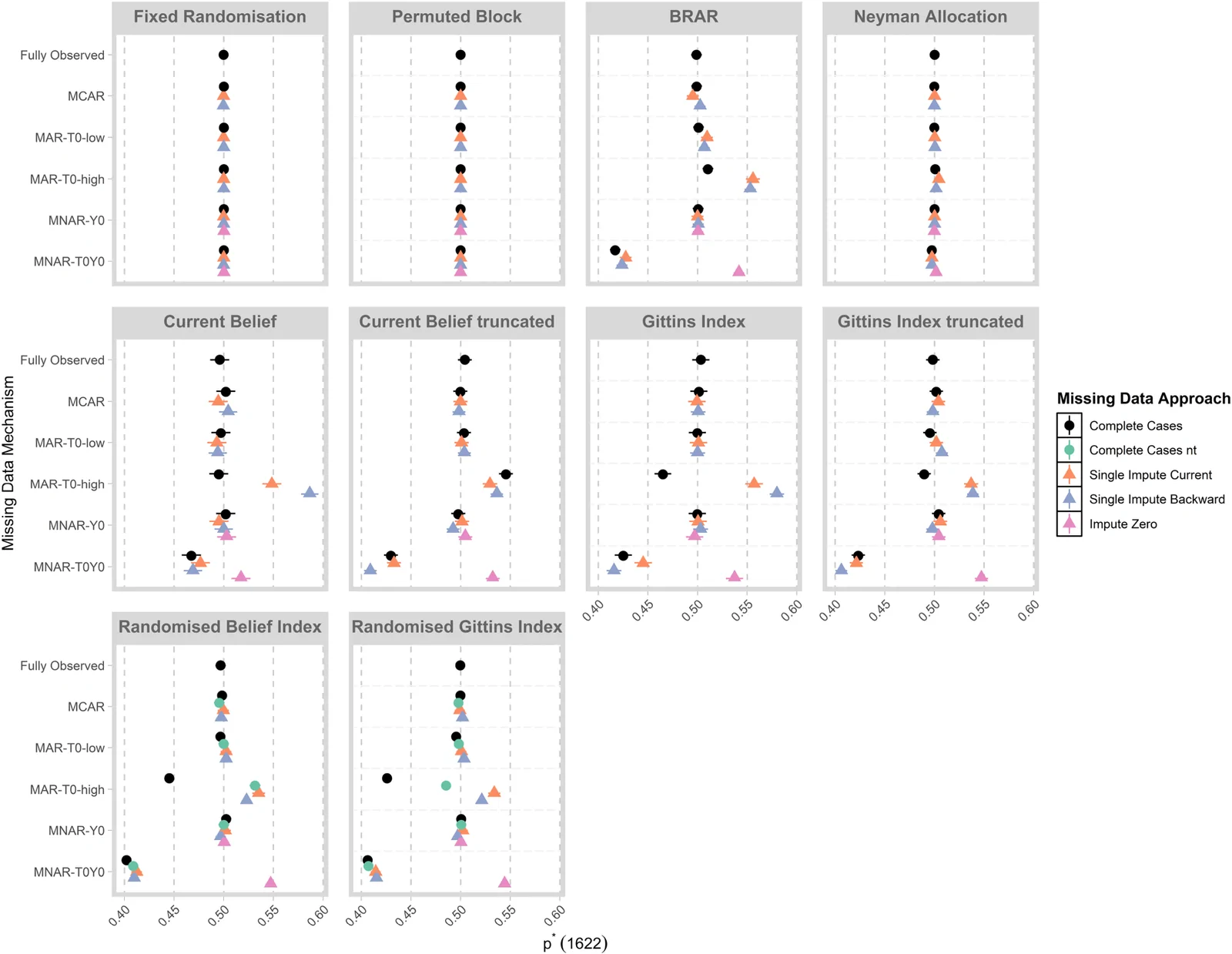
Mean of $p^{*}(1622)$ under the Null scenario for each combination of design, missing data mechanism and missing data strategy. Plots are based on 10,000 simulations and error bars indicate 1.96× MC standard error. *Complete cases-nt*: complete cases for semi-randomised algorithms, where randomised component is given by Equation (6).

**Figure 8.**
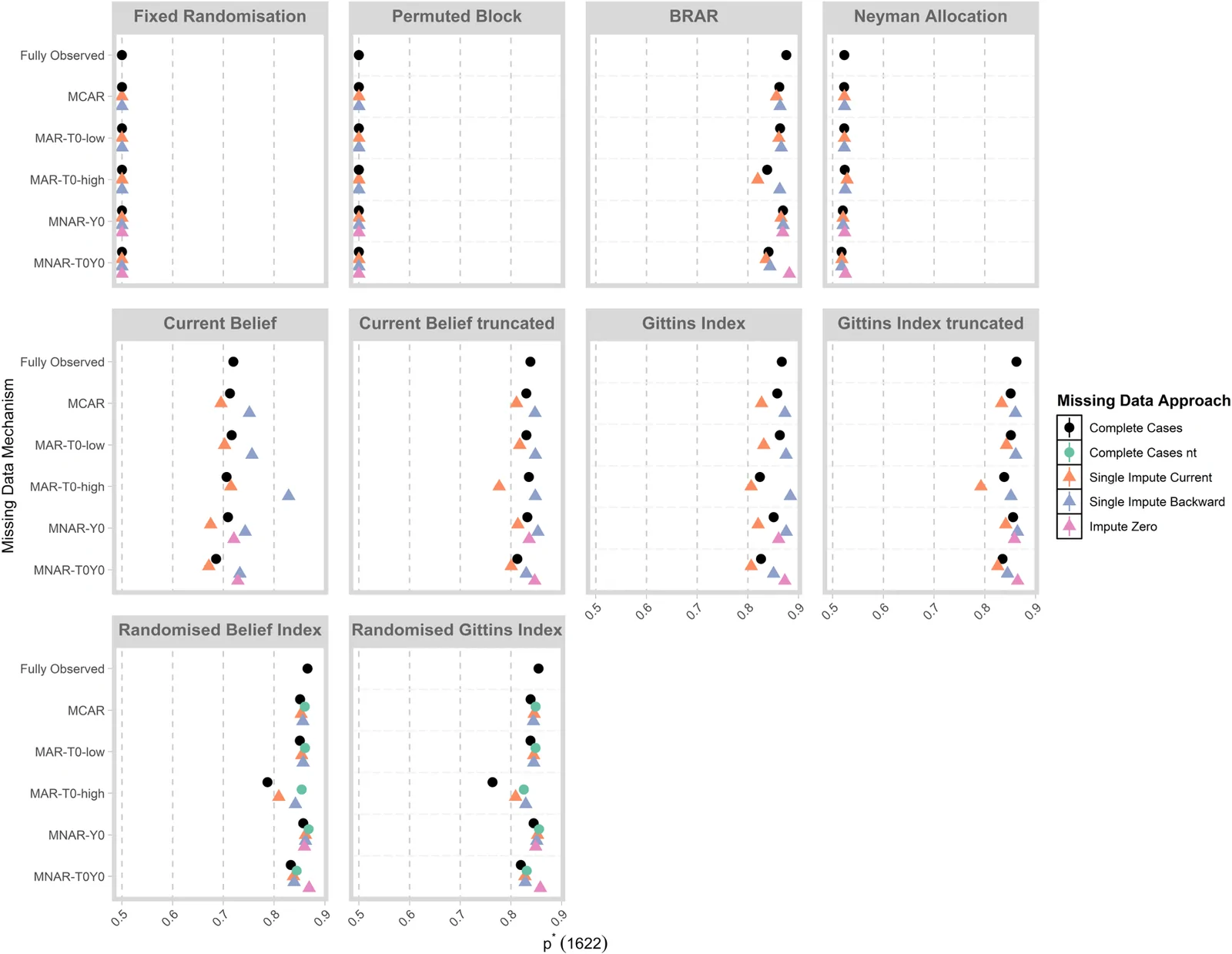
Mean of $p^{*}(1622)$ under the Alternative scenario for each combination of design, missing data mechanism and missing data strategy. Plots are based on 10,000 simulations and error bars indicate 1.96× MC standard error. *Complete cases-nt*: complete cases for semi-randomised algorithms, where randomised component is given by Equation (6).

**Figure 9.**
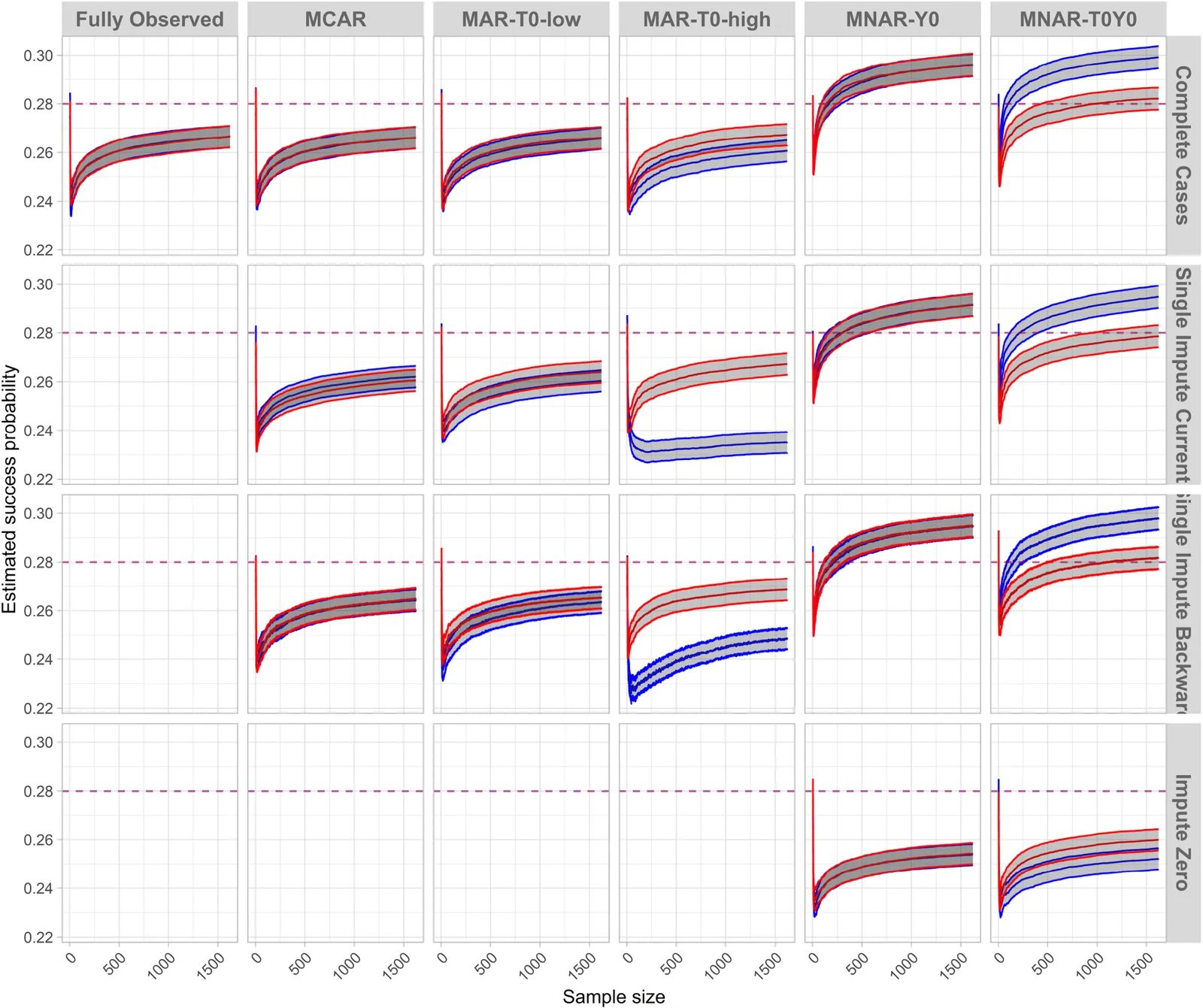
Maximum likelihood estimates of $p_{0}$ (blue) and $p_{1}$ (red) at every sample size are shown for each combination of missing data mechanism (columns) and missing data strategy (rows) when BRAR is used under the Null scenario. The true values are shown by dashed lines. Plots show mean across 10,000 simulations 1.96× Monte Carlo standard error. Note that imputations are used to compute the MLE for single impute current and single impute backward.

**Figure 10.**
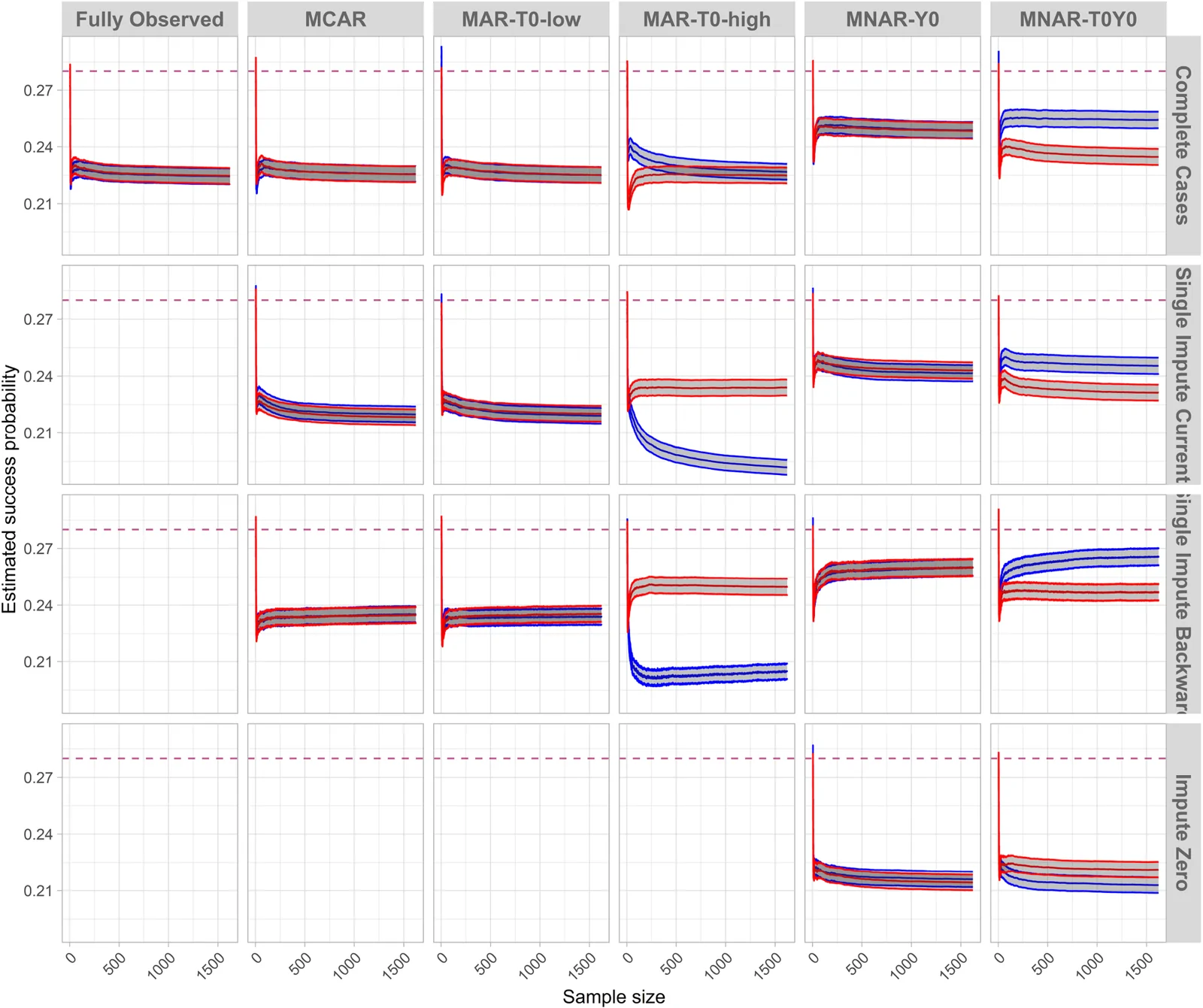
Maximum likelihood estimates of $p_{0}$ (blue) and $p_{1}$ (red) at every sample size are shown for each combination of missing data mechanism (columns) and missing data strategy (rows) when the Gittins index is used under the Null scenario. The true values are shown by dashed lines. Plots show mean across 10,000 simulations 1.96× Monte Carlo standard error. Note that imputations are used to compute the MLE for single impute current and single impute backward.

**Figure 11.**
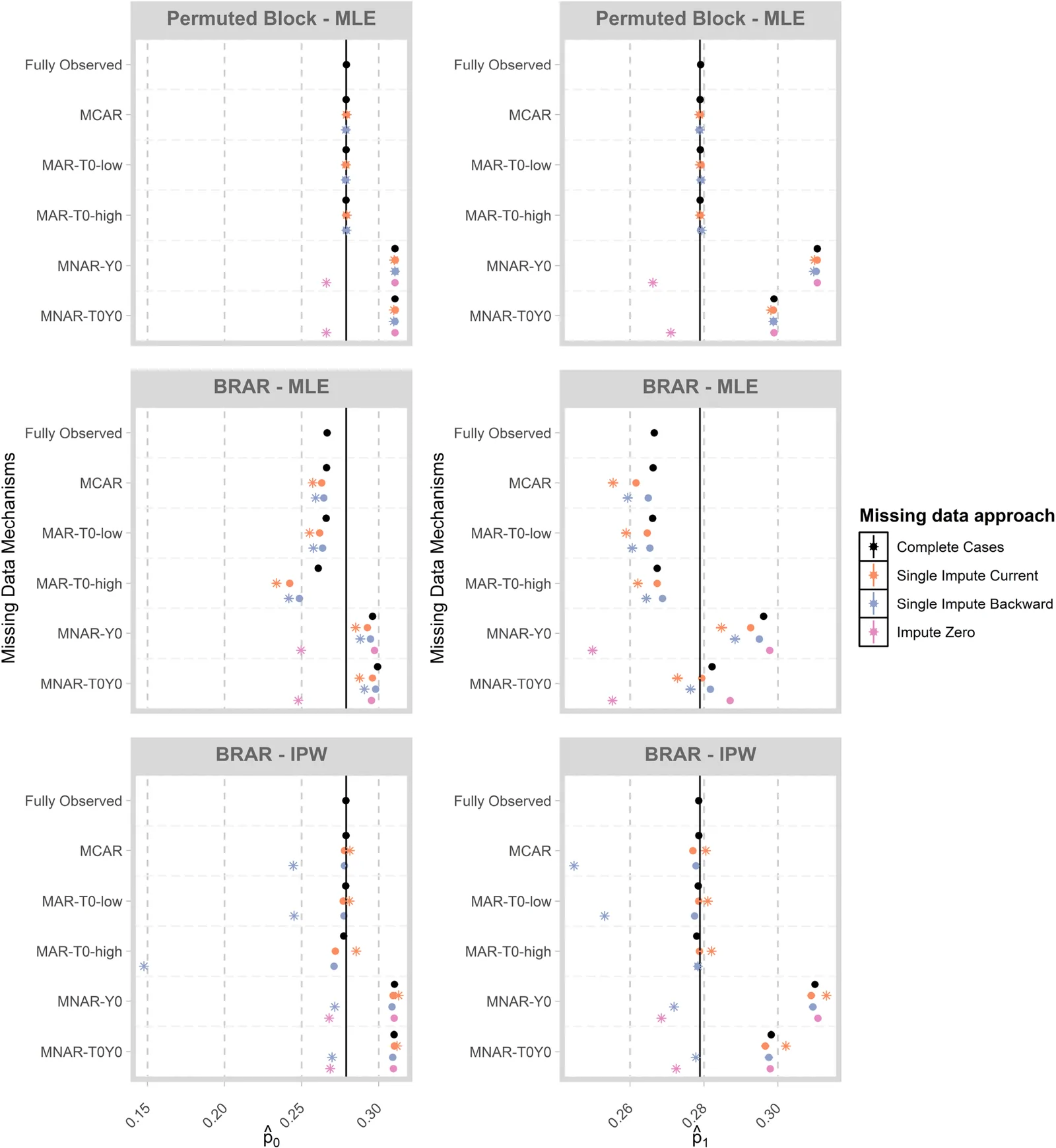
Mean of estimates of $p_{0}$ (left) and $p_{1}$ (right) across 10,000 simulations under the Null scenario when using the MLE for the permuted block design (top), the MLE for BRAR (middle) and the IPW estimator for BRAR (bottom). Error bars indicate 1.96 ± Monte Carlo standard error. For single impute current, single impute backwards and impute zero, the estimate can be computed using complete cases only, shown as a dot, or using both observed and imputed values, shown as a star. In several cases, the two estimates coincide.
