# Supplementary material for: Implementing response-adaptive designs when responses are missing: Impute or ignore?
Source: Stat Methods Med Res. 2025 Aug 29;35(11):2367–88. doi: 10.1177/09622802251366843 (PMC13744590; doi:10.1177/09622802251366843)

Implementing Response-Adaptive Designs when Responses are Missing: Impute or Ignore? Supplementary File 1

Mia S. Tackney, Sofía S. Villar

This file displays additional simulation results for the Null and Alternative scenarios (omitted in the main article and appendix), as well as results for the Null-low and Alternative-low scenarios.

All designs begin with a permuted block of size 4 (two assignments to each arm) where all four responses are observed. This prevents situations where where missing responses at the beginning of the trial lead to extreme imbalance and prevent estimation of success probabilities. In Supplementary File 2, we provide results when this initialisation is not set.

1 Additional results for the Null and Alternative Scenarios

Figure 1: Mean of estimates of p_0 (left) and p_1 (right) across 10,000 simulations under the Null scenario when using the MLE for all designs explored in the simulation study. Error bars, indicate $1.96 \pm$ Monte Carlo standard error. For Single Impute Current, Single Impute Backwards and Impute Zero, the estimate can be computed using complete cases only, shown as a dot, or using both observed and imputed values, shown as a star. In several cases, the two estimates coincide.

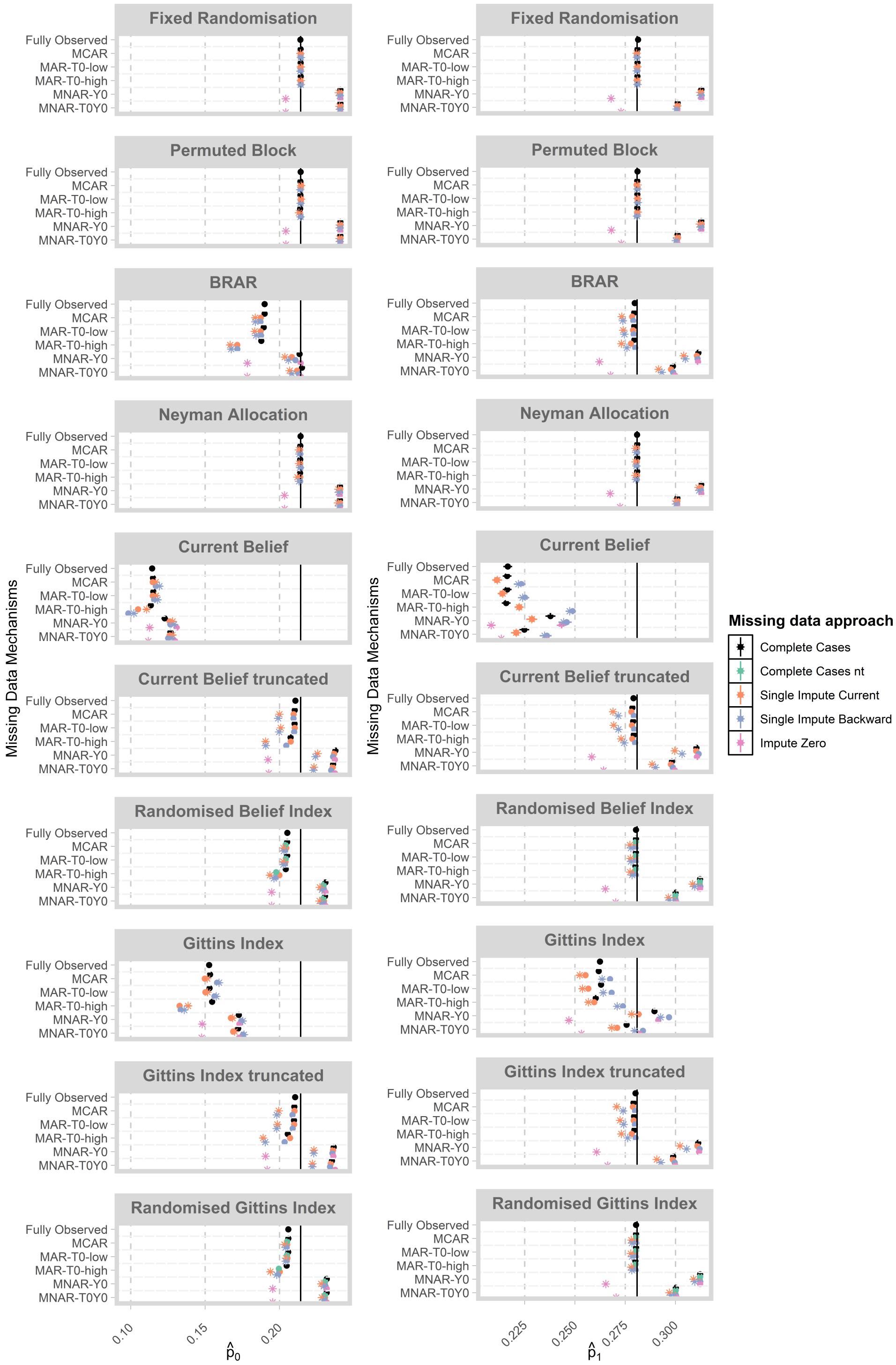

Figure 2: Mean of estimates of p_0 (left) and p_1 (right) across 10,000 simulations under the Alternative scenario when using the MLE for all designs explored in the simulation study. Error bars, indicate $1.96 \pm$ Monte Carlo standard error. For Single Impute Current, Single Impute Backwards and Impute Zero, the estimate can be computed using complete cases only, shown as a dot, or using both observed and imputed values, shown as a star. In several cases, the two estimates coincide.

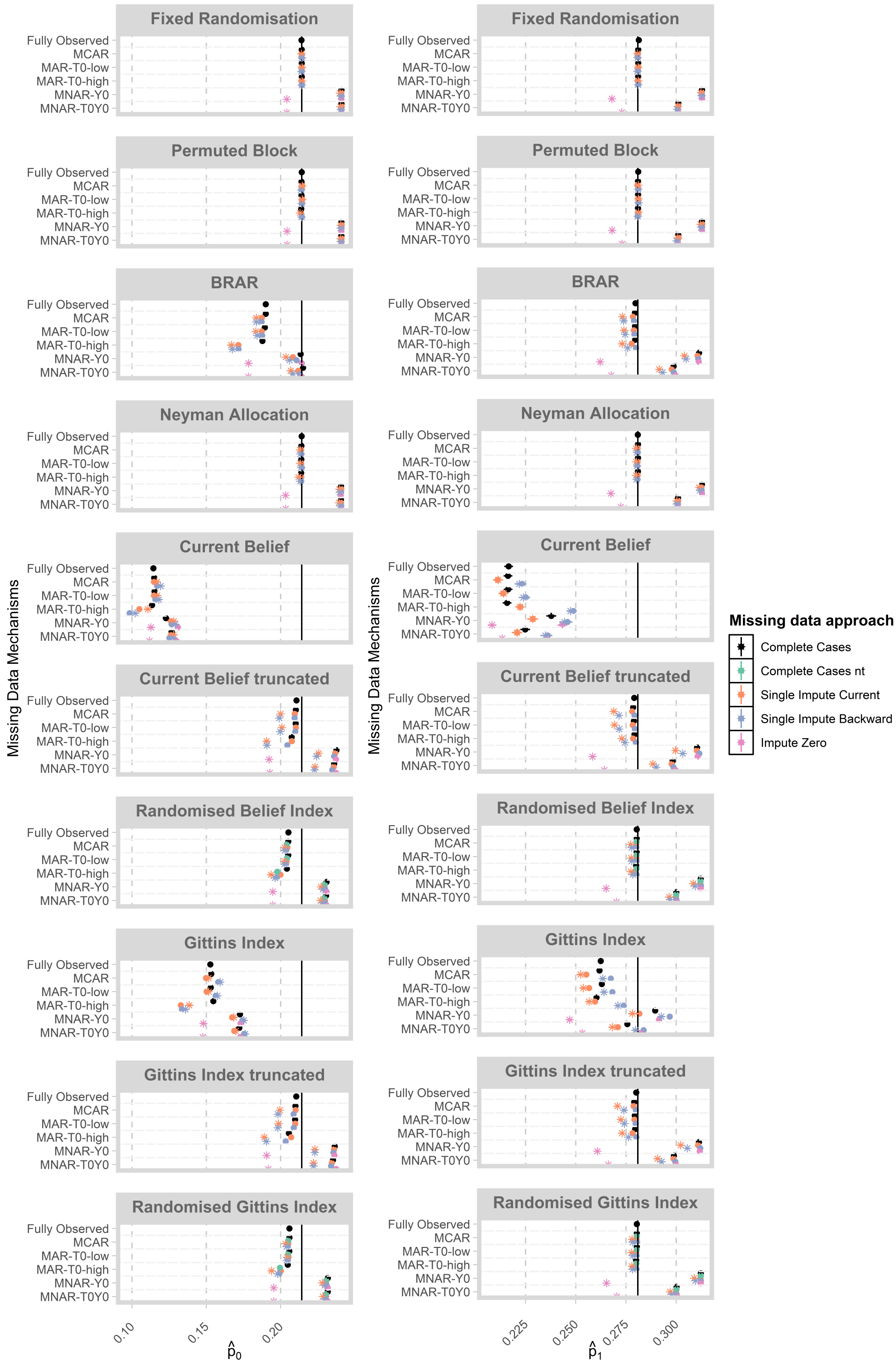

Results for the Null-low scenario

Figure 3: Distribution of $p^*(1622)$ under the Null-low scenario for each combination of design, missing data mechanism and missing data strategy. Plots are based on 10,000 simulations and error bars indicate 1.96× MC standard error. *Complete cases-nt*: complete cases for semi-randomised algorithms, where randomised component is given by Equation (6).

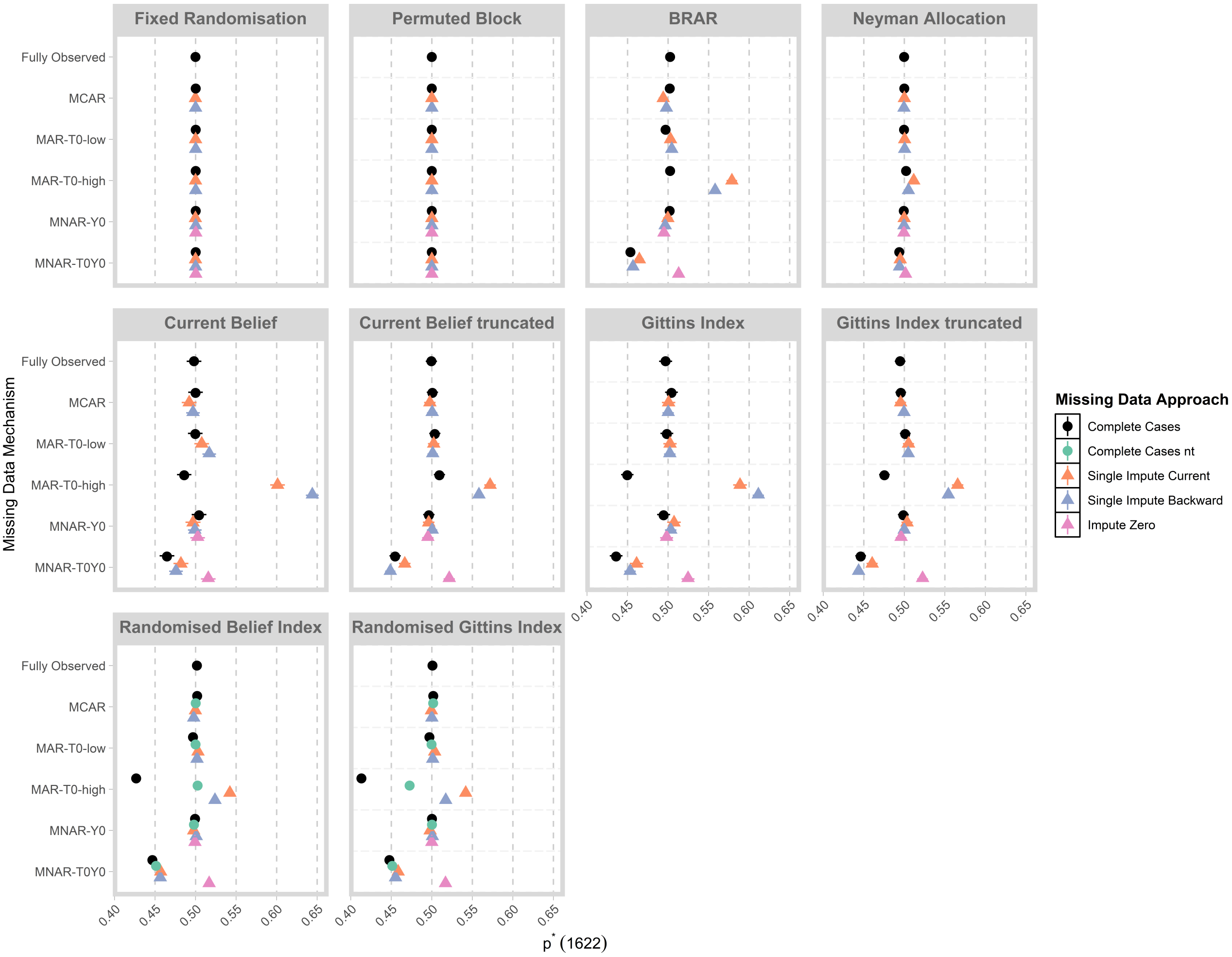

Figure 4: Mean of estimates of p_0 (left) and p_1 (right) across 10,000 simulations under the Null-low scenario when using the MLE for the Permuted Block Design (top), the MLE for BRAR (middle) and the IPW estimator for BRAR (bottom). Error bars, indicate 1.96 ± Monte Carlo standard error. For Single Impute Current, Single Impute Backwards and Impute Zero, the estimate can be computed using complete cases only, shown as a dot, or using both observed and imputed values, shown as a star. In several cases, the two estimates coincide.

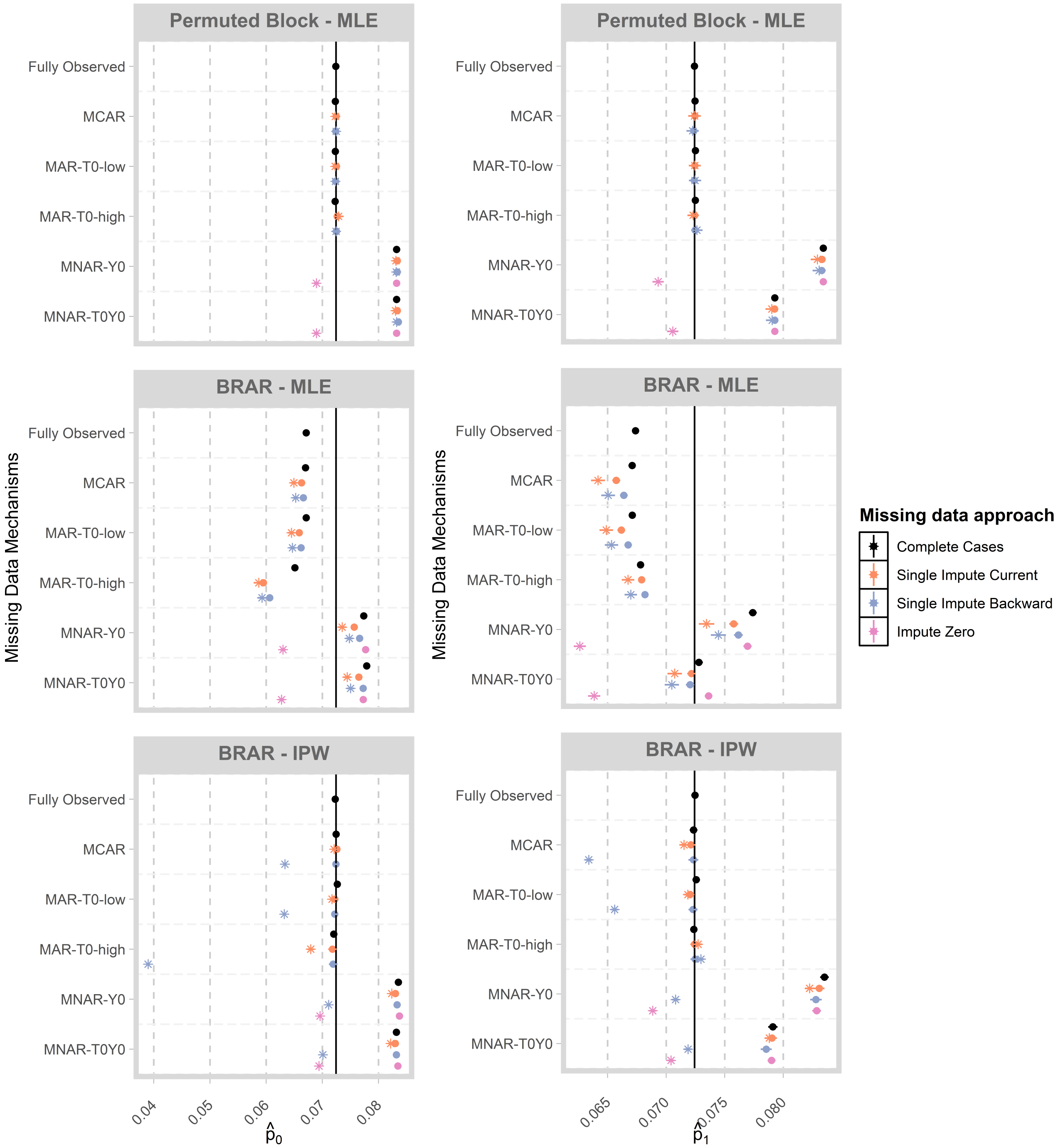

Figure 5: Mean of estimates of p_0 (left) and p_1 (right) across 10,000 simulations under the Null-low scenario when using the MLE for all designs explored in the simulation study. Error bars, indicate 1.96 ± Monte Carlo standard error. For Single Impute Current, Single Impute Backwards and Impute Zero, the estimate can be computed using complete cases only, shown as a dot, or using both observed and imputed values, shown as a star. In several cases, the two estimates coincide.

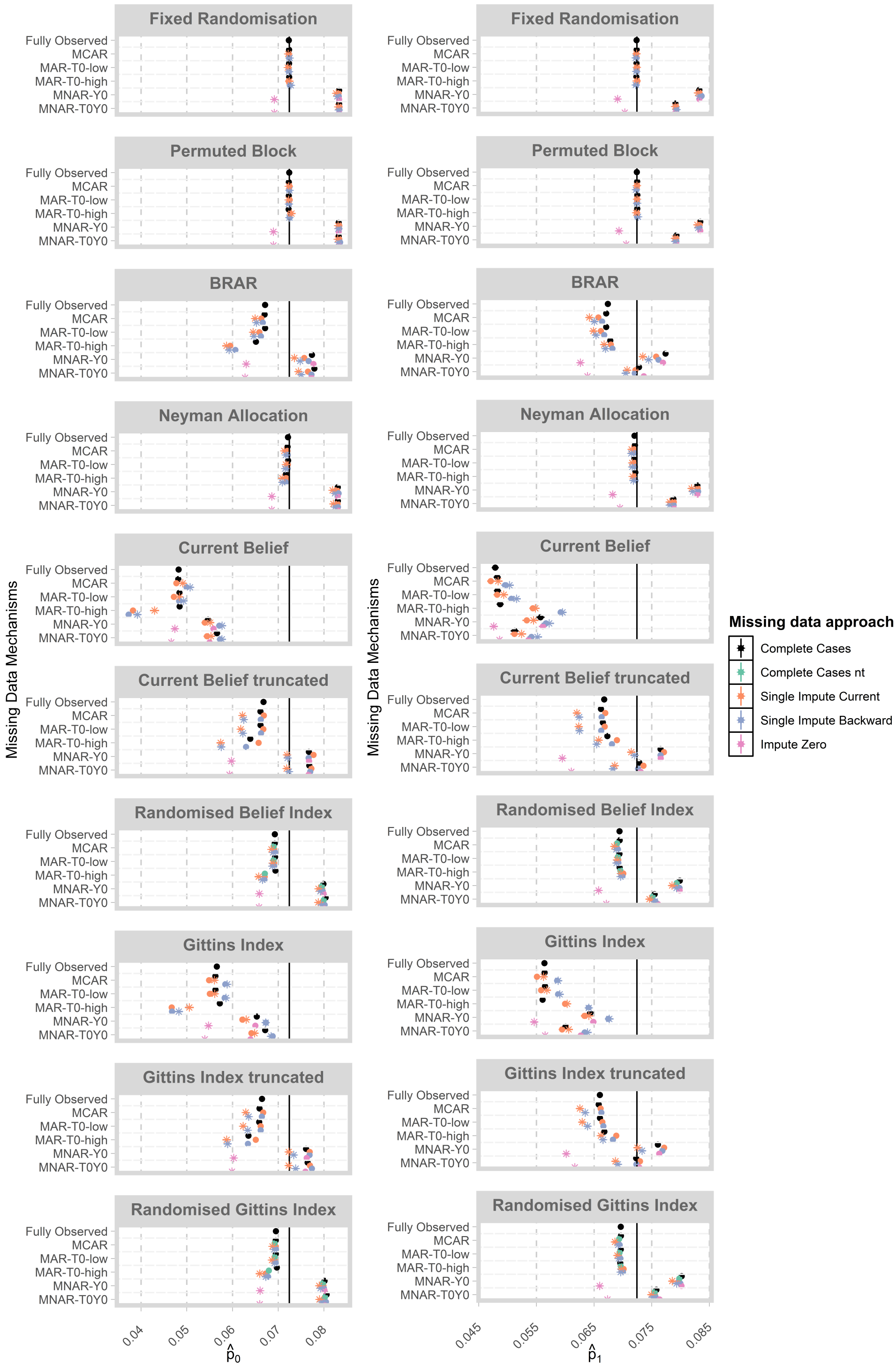

Results for the Alternative-low scenario

Figure 6: Distribution of $p^*(1622)$ under the Alternative-low scenario for each combination of design, missing data mechanism and missing data strategy. Plots are based on 10,000 simulations and error bars indicate 1.96× MC standard error. *Complete cases-nt*: complete cases for semi-randomised algorithms, where randomised component is given by Equation (6).

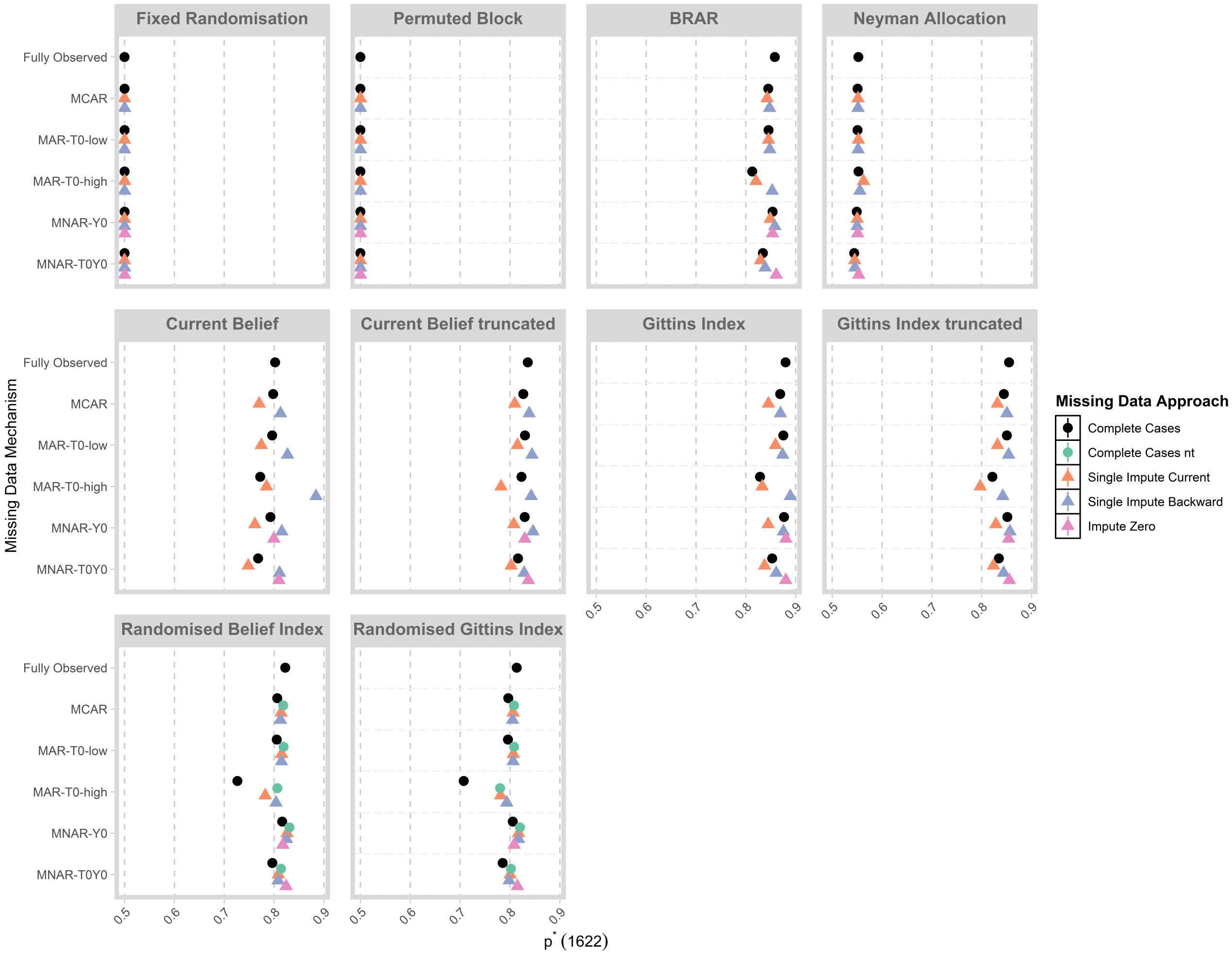

Figure 7: Mean of estimates of p_0 (left) and p_1 (right) across 10,000 simulations under the Alternative-low scenario when using the MLE for the Permuted Block Design (top), the MLE for BRAR (middle) and the IPW estimator for BRAR (bottom). Error bars, indicate 1.96 $\pm$ Monte Carlo standard error. For Single Impute Current, Single Impute Backwards and Impute Zero, the estimate can be computed using complete cases only, shown as a dot, or using both observed and imputed values, shown as a star. In several cases, the two estimates coincide.

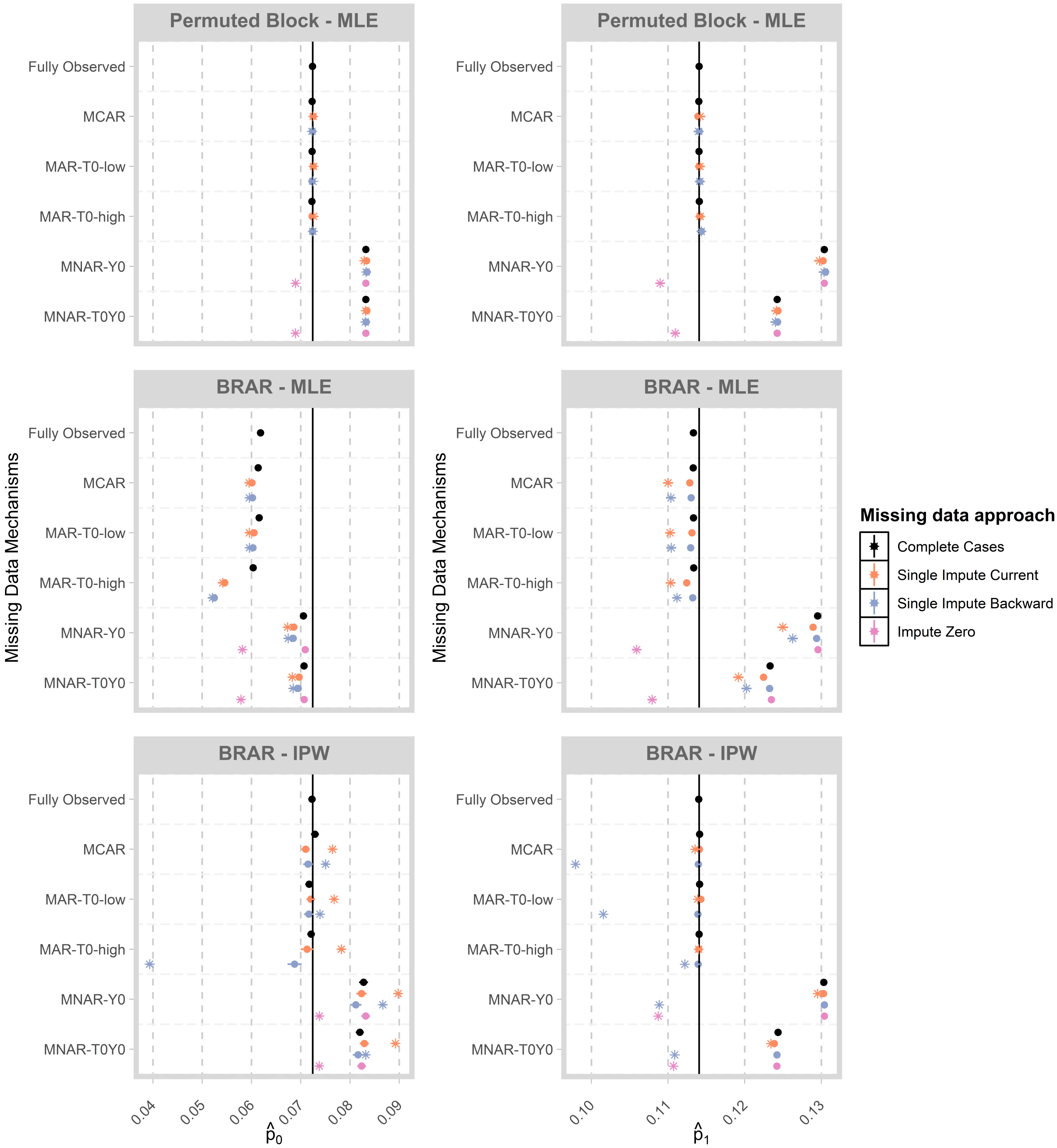

Figure 8: Mean of estimates of p_0 (left) and p_1 (right) across 10,000 simulations under the Alternative-low scenario when using the MLE for all designs explored in the simulation study. Error bars, indicate 1.96 $\pm$ Monte Carlo standard error. For Single Impute Current, Single Impute Backwards and Impute Zero, the estimate can be computed using complete cases only, shown as a dot, or using both observed and imputed values, shown as a star. In several cases, the two estimates coincide.

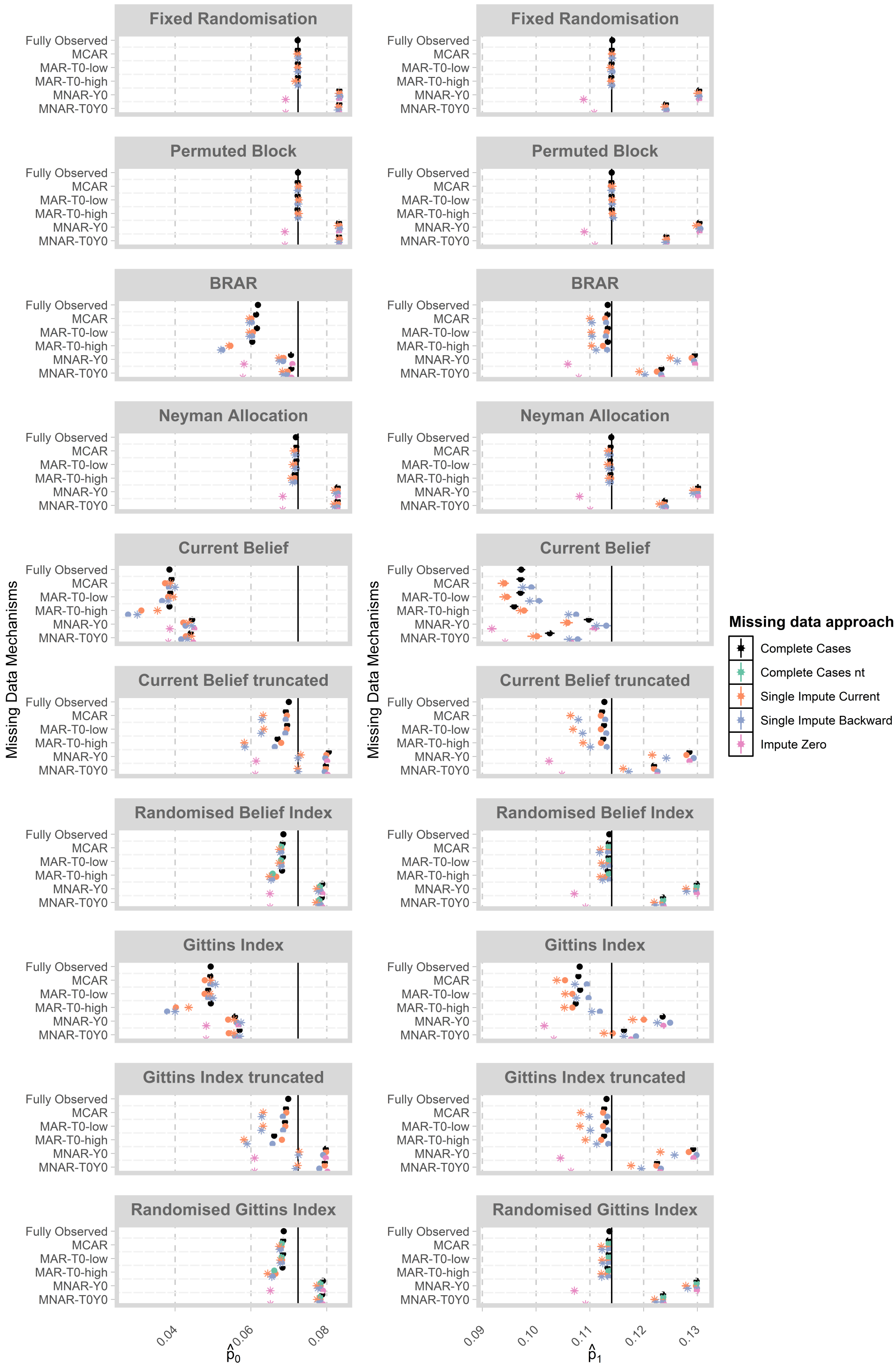

Supplement: Supplementary file 1 [file sj-pdf-1-smm-10.1177_09622802251366843.pdf]
