# Supplementary material for: Implementing response-adaptive designs when responses are missing: Impute or ignore?
Source: Stat Methods Med Res. 2025 Aug 29;35(11):2367–88. doi: 10.1177/09622802251366843 (PMC13744590; doi:10.1177/09622802251366843)

Implementing Response-Adaptive Designs when Responses are Missing: Impute or Ignore? Supplementary File 2

Mia S. Tackney, Sofía S. Villar

In the results shown in the main article and Supplementary File 1, all designs begin with a permuted block of size 4 (two assignments to each arm) where all four responses are observed. This prevents situations where where missing responses at the beginning of the trial lead to extreme imbalance and prevent estimation of success probabilities.

In this file, we present simulation results for the Null and Alternative scenarios where this initialisation is **not** set.

Design	Missing Data Mechanism	Data Generating Mechanism	Missingness Strategy	No. reps $\hat{p}_0 = NA$	No. reps $\hat{p}_1 = NA$
Gittins Index	MAR-T0-high	Null	Single Impute Current	21	0
Gittins Index	MAR-T0-high	Alternative	Single Impute Current	32	0
Gittins Index	MAR-T0-high	Null	Single Impute Backward	20	0
Gittins Index	MAR-T0-high	Alternative	Single Impute Backward	22	0
Current Belief	MCAR	Null	Single Impute Current	23	25
Current Belief	MCAR	Alternative	Single Impute Current	30	3
Current Belief	MAR-T0-low	Null	Single Impute Current	24	12
Current Belief	MAR-T0-low	Alternative	Single Impute Current	22	2
Current Belief	MAR-T0-high	Null	Single Impute Current	470	2
Current Belief	MAR-T0-high	Alternative	Single Impute Current	487	0
Current Belief	MCAR	Null	Single Impute Backward	14	7
Current Belief	MCAR	Alternative	Single Impute Backward	11	0
Current Belief	MAR-T0-low	Null	Single Impute Backward	8	7
Current Belief	MAR-T0-low	Alternative	Single Impute Backward	16	0
Current Belief	MAR-T0-high	Null	Single Impute Backward	471	0
Current Belief	MAR-T0-high	Alternative	Single Impute Backward	469	0
Current Belief	MNAR-Y0	Null	Single Impute Current	40	39
Current Belief	MNAR-Y0	Alternative	Single Impute Current	38	6
Current Belief	MNAR-T0Y0	Null	Single Impute Current	29	18
Current Belief	MNAR-T0Y0	Alternative	Single Impute Current	30	2
Current Belief	MNAR-Y0	Null	Single Impute Backward	20	20
Current Belief	MNAR-Y0	Alternative	Single Impute Backward	36	2
Current Belief	MNAR-T0Y0	Null	Single Impute Backward	18	9
Current Belief	MNAR-T0Y0	Alternative	Single Impute Backward	22	0
Current Belief	MNAR-Y0	Null	Impute Zero	37	35
Current Belief	MNAR-Y0	Alternative	Impute Zero	49	1
Current Belief	MNAR-T0Y0	Null	Impute Zero	37	9
Current Belief	MNAR-T0Y0	Alternative	Impute Zero	29	1

Table 1: We report the number of repetitions in simulations (out of a total of 10,000) which do not provide a valid estimate for p_0 or p_1. This is due to an early selection and extreme imbalance in treatment arms, which occurs for Gittins Index and Current Belief.

Results for the Null scenario

Figure 1: Distribution of $p^*(1622)$ under the Null scenario for each combination of design, missing data mechanism and missing data strategy. Plots are based on 10,000 simulations and error bars indicate $1.96\times$ MC standard error. *Complete cases-nt*: complete cases for semi-randomised algorithms, where randomised component is given by Equation (6).

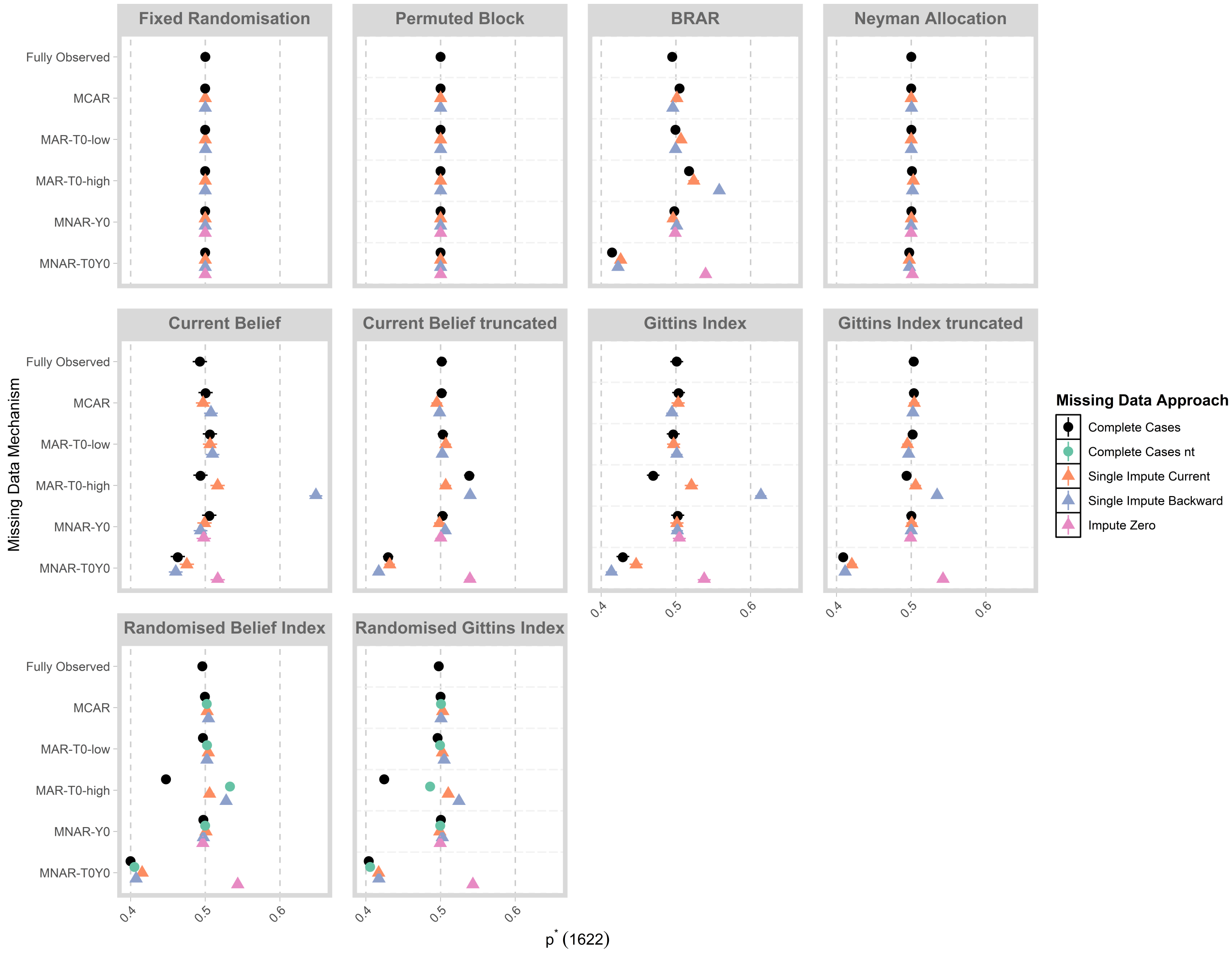

Figure 2: Mean of estimates of p_0 (left) and p_1 (right) across 10,000 simulations under the Null scenario when using the MLE for the Permuted Block Design (top), the MLE for BRAR (middle) and the IPW estimator for BRAR (bottom). Error bars, indicate 1.96 $\pm$ Monte Carlo standard error. For Single Impute Current, Single Impute Backwards and Impute Zero, the estimate can be computed using complete cases only, shown as a dot, or using both observed and imputed values, shown as a star. In several cases, the two estimates coincide.

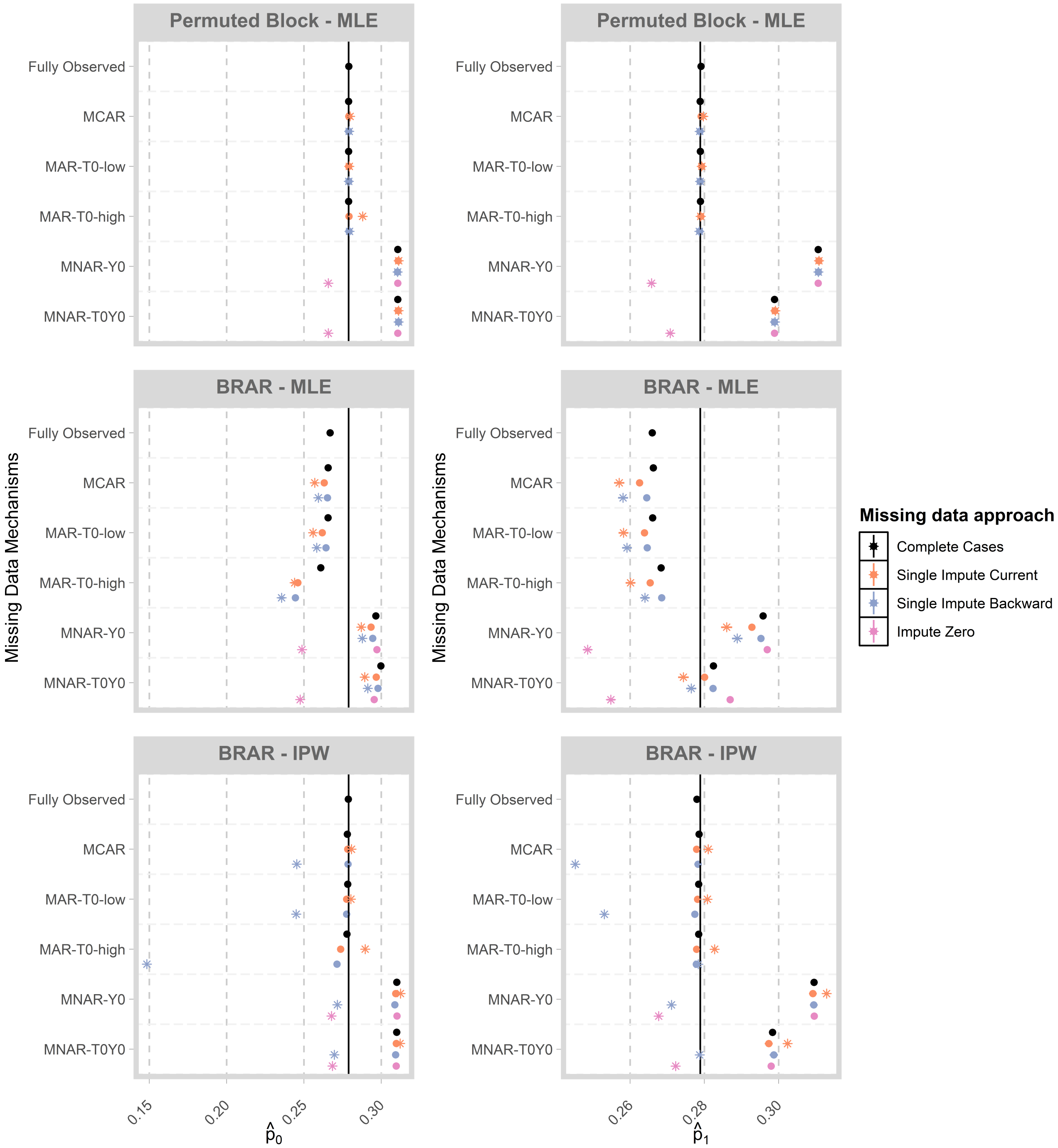

Figure 3: Mean of estimates of p_0 (left) and p_1 (right) across 10,000 simulations under the Null scenario when using the MLE for all designs explored in the simulation study. Error bars, indicate 1.96 ± Monte Carlo standard error. For Single Impute Current, Single Impute Backwards and Impute Zero, the estimate can be computed using complete cases only, shown as a dot, or using both observed and imputed values, shown as a star. In several cases, the two estimates coincide.

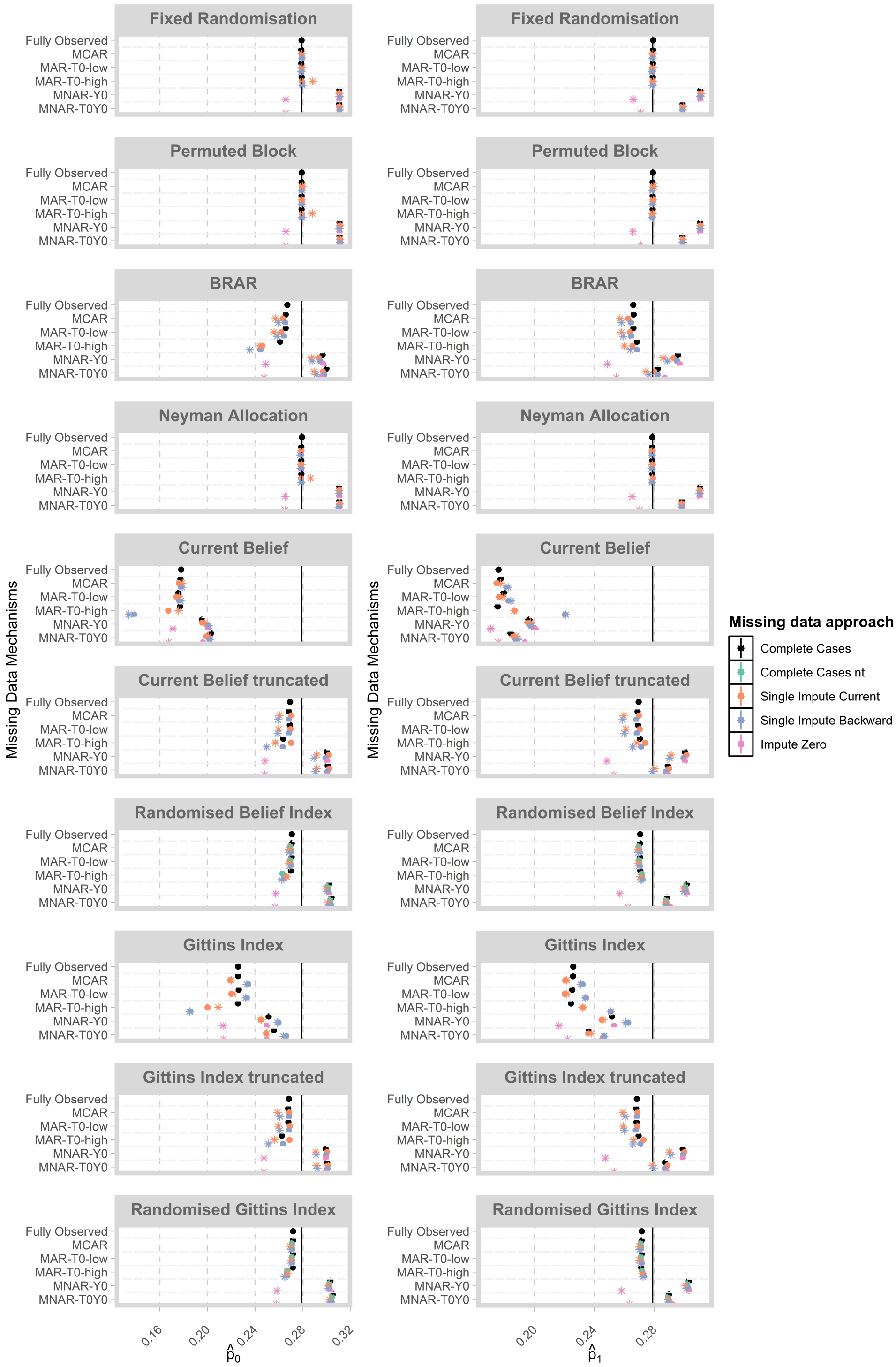

Results for the Alternative scenario

Figure 4: Distribution of $p^*(1622)$ under the Alternative scenario for each combination of design, missing data mechanism and missing data strategy. Plots are based on 10,000 simulations and error bars indicate 1.96× MC standard error. *Complete cases-nt*: complete cases for semi-randomised algorithms, where randomised component is given by Equation (6).

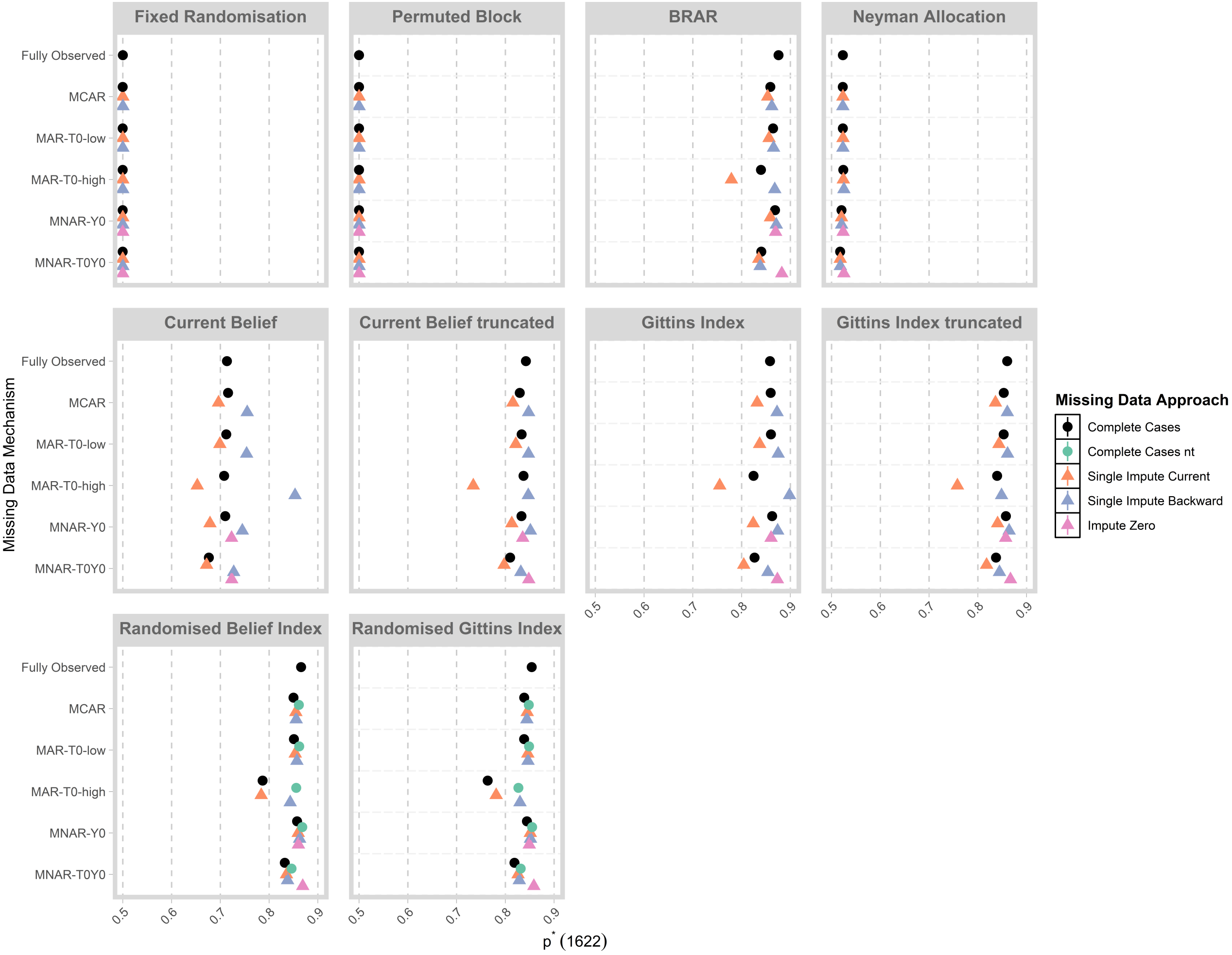

Figure 5: Mean of estimates of p_0 (left) and p_1 (right) across 10,000 simulations under the Alternative scenario when using the MLE for the Permuted Block Design (top), the MLE for BRAR (middle) and the IPW estimator for BRAR (bottom). Error bars, indicate 1.96 $\pm$ Monte Carlo standard error. For Single Impute Current, Single Impute Backwards and Impute Zero, the estimate can be computed using complete cases only, shown as a dot, or using both observed and imputed values, shown as a star. In several cases, the two estimates coincide.

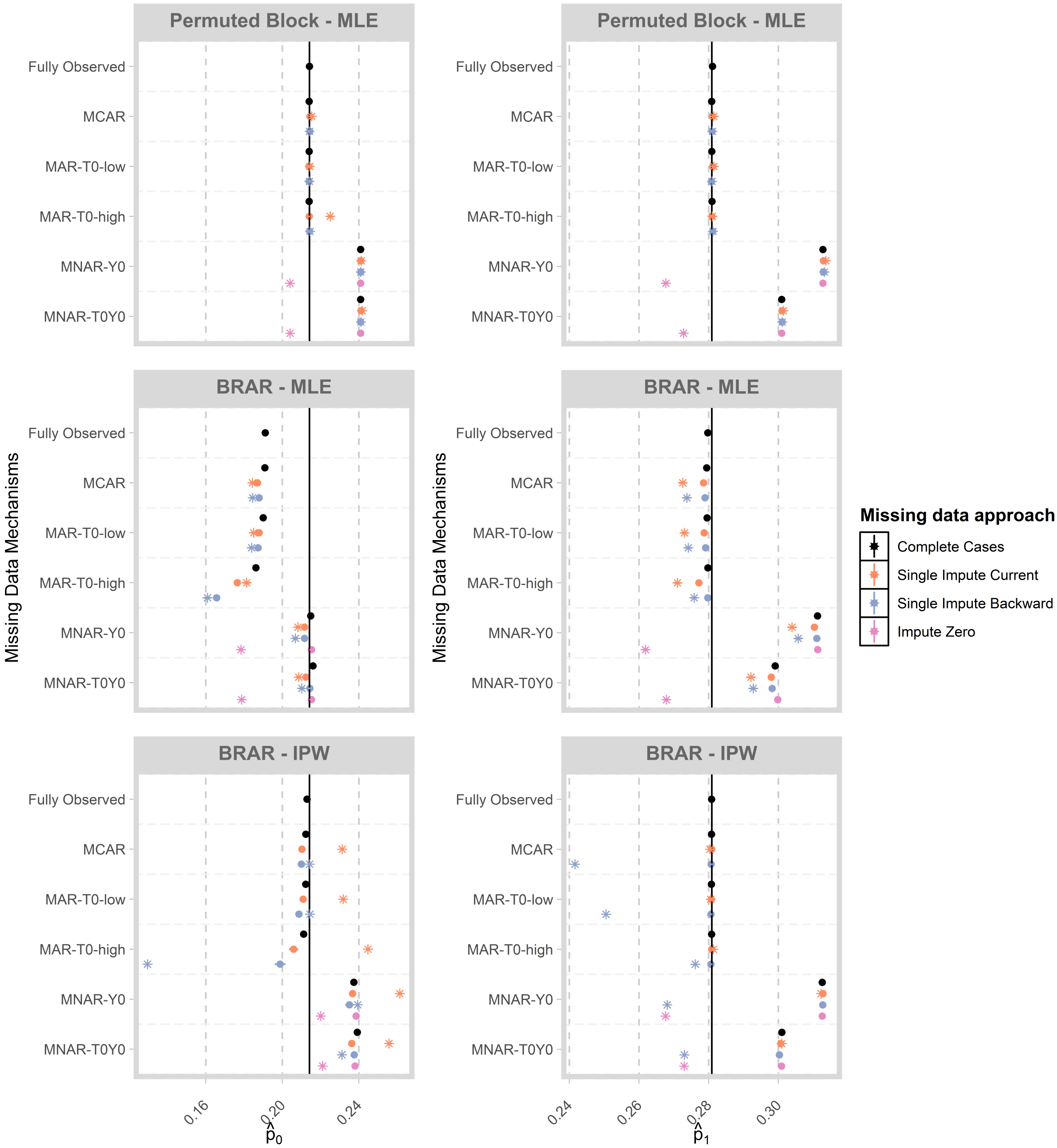

Figure 6: Mean of estimates of p_0 (left) and p_1 (right) across 10,000 simulations under the Alternative scenario when using the MLE for all designs explored in the simulation study. Error bars, indicate $1.96 \pm$ Monte Carlo standard error. For Single Impute Current, Single Impute Backwards and Impute Zero, the estimate can be computed using complete cases only, shown as a dot, or using both observed and imputed values, shown as a star. In several cases, the two estimates coincide.

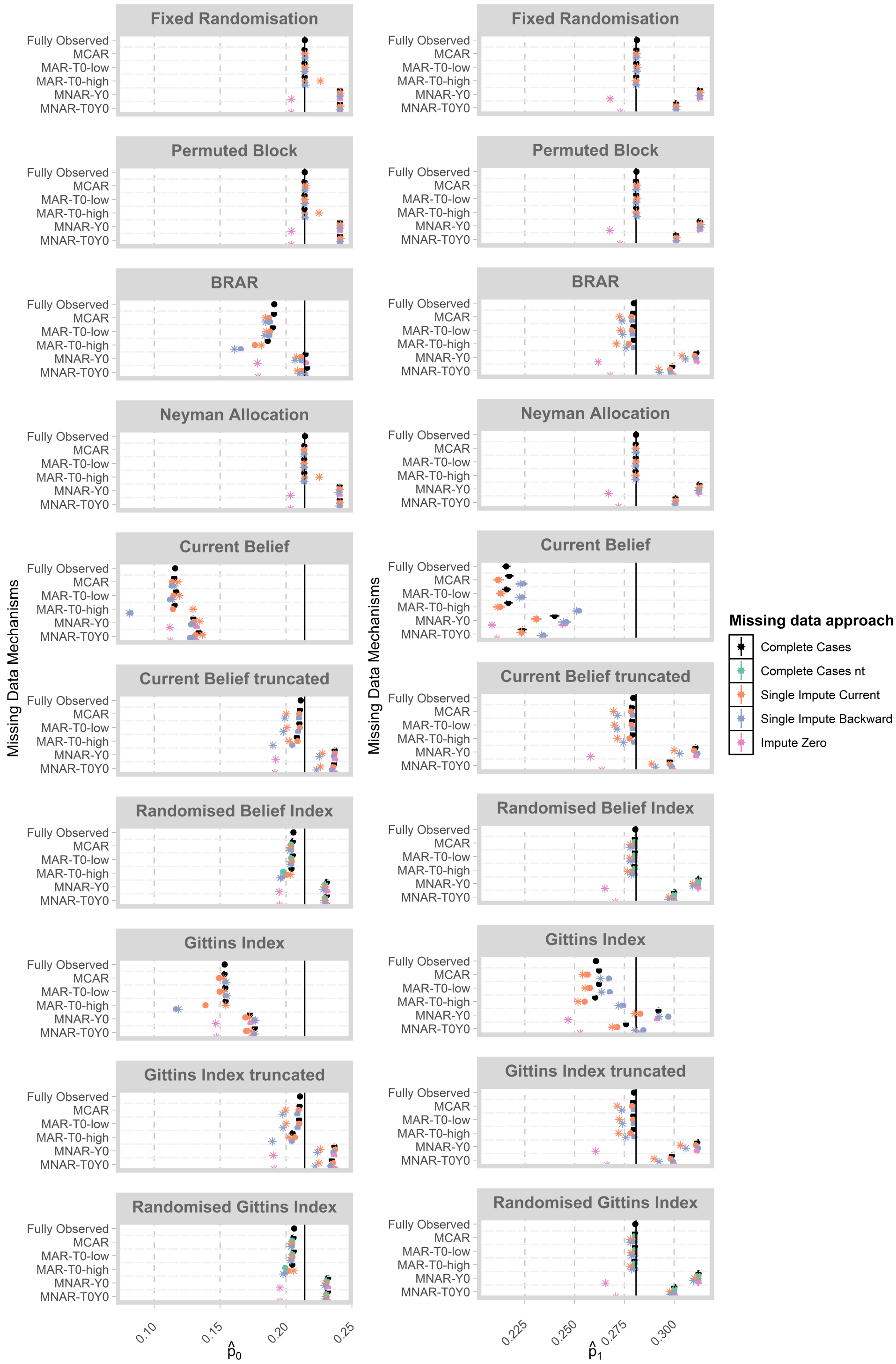

Supplement: Supplementary file 2 [file sj-pdf-2-smm-10.1177_09622802251366843.pdf]
